# Evaluating the Effects of Flavonoids on Insects: Implications for Managing Pests Without Harming Beneficials

**DOI:** 10.3390/insects15120956

**Published:** 2024-12-01

**Authors:** Eric Wellington Riddick

**Affiliations:** Biological Control of Pests Research Unit, Agricultural Research Service, United States Department of Agriculture, Stoneville, MS 38776, USA; eric.riddick@usda.gov; Tel.: +1-662-686-3646

**Keywords:** chemical ecology, flavonoid-insect interactions, herbivores, insect health, integrated pest management, pollinators

## Abstract

Flavonoids have multiple functions, including host-plant defense against attacks from herbivorous insects. This manuscript reviewed and analyzed the scientific literature to test the hypothesis that flavonoids can be utilized to manage pests without causing significant harm to beneficials. The methodology involved using recognized literature databases, e.g., Web of Science, Scopus, and CAB Abstracts, via the USDA-ARS, National Agricultural Library, DigiTop literature retrieval system. Data were compiled in tables and subjected to statistical analysis, when appropriate. Flavonoids were generally harmful to true bugs and true flies but harmless to honey bees. Flavonoid glycosides showed a tendency to harm true bugs (Heteroptera) and true flies (Diptera). Flavonoid glycosides were harmless to sawflies. Flavonoids and flavonoid glycosides produced a mixture of harmful and harmless outcomes to herbivorous beetles, depending on the species. Flavonoid glycosides were harmless to butterflies. In conclusion, specific flavonoids could function as feeding stimulants or deterrents, oviposition stimulants or deterrents, chemical protectants from pesticides, mating attractants, less-toxic insecticides, and other functions. Flavonoids could manage some insect pests without causing significant harm to beneficials (e.g., honey bees). Flavonoid-based insecticides could serve as environmentally benign alternatives to broad-spectrum insecticides against some pests, but field testing is necessary.

## Simple Summary

Flavonoids are secondary metabolites that deter attacks from some plant-feeding insects. The hypothesis that flavonoids and flavonoid glycosides can be utilized to manage pest insects without negatively affecting beneficial insects was evaluated. Scientific literature databases were examined. Flavonoids were harmful to most true bugs and true flies but harmless to bees. Flavonoid glycosides showed a tendency to harm true bugs and true flies but not harm sawflies. Flavonoids and flavonoid glycosides caused a mixture of harmful and harmless outcomes to plant-feeding beetles. Flavonoid glycosides were harmless to butterflies. In conclusion, these compounds have moderate potential as attractants, stimulants, repellents, deterrents, and less-toxic insecticides against some pests, especially true bugs and true flies, without harming beneficials.

## 1. Introduction

### 1.1. Flavonoid–Insect Interactions

Plant secondary metabolites can be divided into broad groupings, such as terpenoids, alkaloids, and phenolics; phenolics (polyphenols) are the most diverse and widely distributed amongst vascular and non-vascular plant species [1]. Polyphenols include phenolic acids, stilbenes, lignans, and flavonoids. Flavonoids are abundant in crop and non-crop plants, plant products, i.e., pollen, fruits, and seeds, and represent over 4500 distinct compounds [2,3,4]. They exist in plant tissues in a free form (aglycones) or conjugated with sugar molecules, i.e., glycosides [5]. Flavonoids possess a 15-carbon flavone structure, C6-C3-C6, with two benzene rings linked by a three-carbon pyran ring. The molecular structure and nomenclature of different flavonoids have been described and illustrated elsewhere [6,7,8]. They can be categorized into major classes, i.e., flavonols, flavones, flavanones, flavan-3-ols, isoflavones, and anthocyanins. Flavonols and flavones are found in almost all plants, primarily in foliage [9]. In plants, flavonols are often more abundant than flavones.

Theories on the origin of flavonoids and their role in plant development and responses to environmental stimuli have been proposed. The hypothesis that flavonol molecules were intimately involved in the evolution of early terrestrial plants such as mosses and liverworts has been proposed [10]. Flavonoids have diverse functions in plants. For example, some have been responsible for the color and fragrance of flowers. Some have regulated plant cell growth, responded to abiotic stress, such as UV radiation and high temperatures, and protected plants from insect pests and pathogens [3,8,11,12,13,14].

Knowledge of the diversity, molecular structures, and functions of flavonoids has accumulated in the literature. However, the role of these compounds in insect-plant interactions has not been fully defined [15,16]. Moreover, relatively few of these compounds have been examined for their potential to curb pests and encourage beneficials in agricultural and non-agricultural landscapes. Some evidence suggests that flavonoids could function as less-toxic insecticides. More than 280 different flavonoid compounds have been studied for their potential pesticidal activity [17,18]. Additionally, the capacity of flavonoids to ameliorate the toxic effects of synthetic (conventional) pesticides on pollinators, including the western honey bee *Apis mellifera* L. (Order Hymenoptera: Family Apidae) has been implicated [19].

Evidence that insects discriminate amongst flavonoids has been published. However, the mechanism of action of these compounds in influencing insect feeding, oviposition, and reproduction has not been elucidated [15]. A review of the literature on one of the most abundant and widely studied flavonoids, i.e., quercetin, demonstrated multiple functions such as protecting crop plants from some herbivores (moths, beetles, and true bugs), but attracting and stimulating feeding behavior in others (which have adapted to feeding on host plants) and extending the lifespan of pollinators (honey bees) [20].

The aim of this manuscript is to review the literature on flavonoid-insect interactions and highlight the effects of flavonoids (and flavonoid glycosides) on beneficial and pest insects, many of which are important to agriculture. Knowledge of flavonoid multifunctionalities could foster management practices that reduce agricultural pests without affecting pollinators and natural enemies.

### 1.2. Hypothesis and Methodology

The hypothesis that flavonoids can be utilized to manage pests without causing significant harm to beneficials was tested. Nearly all flavonoids and flavonoid glycosides (with attached sugar molecules), affecting or not affecting insects, have been listed in Tables S1 and S2, respectively, in the Supplementary Materials. Methodology involved reviewing all available peer-reviewed literature pertaining to flavonoid-insect interactions using the USDA, National Agricultural Library (NAL), DigiTop literature retrieval system (https://digitop.nal.usda.gov, 12 August 2023). DigiTop provided access to scientific databases such as Web of Science, SCOPUS, AGRICOLA, CABI, Zoological Records, and others. The taxa covered in this manuscript included butterflies (Lepidoptera), bees (Hymenoptera), sawflies (Hymenoptera), beetles (Coleoptera), true bugs (Hemiptera), and true flies (Diptera). Other taxa, such as moths (Lepidoptera), grasshoppers and crickets (Orthoptera), and termites (Isoptera), were not included in this text. Evidence that flavonoids and flavonoid glycosides had positive, negative, or neutral effects on insect behavior and life history parameters was compiled into Tables S3 and S4, respectively, in the Supplementary Materials. Positive effects improved the health (feeding, growth, development, oviposition, reproduction, survival, mating, etc.), negative effects reduced the health, and neutral effects had no impact on the health of insects. Next, these results were grouped into harmless (positive and neutral effects combined) and harmful (negative effects) outcomes and analyzed using a *Z*-test for proportional data (SigmaPlot^®^ for Windows, version 15, 2023). Note that artificial intelligence (AI) technologies were not used in the review of the literature, data analysis, or writing of this manuscript.

This work could suggest which flavonoids or flavonoid glycosides could be isolated from plants (or plant products) and formulated into stimulants, attractants, repellents, deterrents, and less-toxic insecticides to manage pests of agricultural crops. It is envisioned that flavonoid (and flavonoid glycoside) formulations could be improved via nanotechnology to increase time of persistence in field applications. The identification of alternative and abundant sources of plant flavonoids, such as agricultural wastes, e.g., citrus peels [21], is crucial to the broad-scale application of flavonoids in integrated pest management programs.

## 2. Effects of Flavonoids on Insects

### 2.1. Butterflies (Order Lepidoptera)

Butterflies are significant pollinators of native plants near agricultural landscapes [22,23]. Less well-documented are the effects of plant flavonoids on their life history, behavior, and ecology. The role of chemical cues from host plants in oviposition behavior of the black swallowtail butterfly *Papilio polyxenes* F. (Order Lepidoptera: Family Papilionidae) has been discovered [24]. Larvae have adapted to feeding on plants containing high levels of prooxidant allelochemicals. Researchers tested whether antioxidant enzymes of *P*. *polyxenes* could be induced (activated) by a challenge from a common prooxidant (quercetin), by dipping parsley *Petroselinum crispum* (Mill.) (Family Umbelliferae) leaves, its rearing host plant, in solutions of quercetin and then monitoring antioxidant enzyme activities in larvae [25]. Results indicated that superoxide dismutase activity increased nearly two-fold at the maximum quercetin test concentration (2.0%, w/w). In contrast, glutathione reductase activity and catalase activity decreased as quercetin concentration increased. Moreover, glutathione reductase activity was completely inhibited at the maximum quercetin concentration (2.0%, w/w). These results suggest that quercetin had a positive effect on superoxide dismutase activity but a negative effect on glutathione reductase and catalase activity in *P*. *polyxenes*.

*Papilio polyxenes* females were stimulated to oviposit onto surfaces via tarsal contact with wild carrot *Daucus carota* L. (Family Umbelliferae) leaf extracts containing trans-chlorogenic acid and luteolin 7-O-(6″-O-malonyl)-β-D-glucopyranoside [26]. Either compound acting alone did not stimulate oviposition, but combining the two elicited an oviposition response representing approximately 70% of the response to the original extract [26]. Therefore, combining these two compounds had a positive effect on *P*. *polyxenes* oviposition behavior.

The monarch butterfly *Danaus plexippus* (Family Nymphalidae) is well known for its spectacular migration to overwintering sites each year [27]. Endogenous quercetin bound with glucosides or glycosides stimulated oviposition behavior of *D*. *plexippus* females on the host plant *Asclepias curassavica* L. [28]. Of the seven quercetin glycosides isolated from *A*. *curassavica* leaves, the two quercetin dirhamnosyl glycosides, the rutinoside, and the glucosylgalactose were active oviposition stimulants, at 0.5 g leaf equivalents, as females made tarsal contact with treated surfaces in laboratory bioassays. Oviposition stimulation was significantly greater when all active quercetin glycosides were combined than when they were separated individually [28].

The zebra swallowtail butterfly *Eurytides marcellus* (Cramer) (Family Papilionidae) is distributed in eastern North America, and females depend on a single compound to stimulate oviposition onto one of its host plants, the American pawpaw tree *Asimina triloba* (L.) Dunal (Family Annonaceae) [29,30]. Larvae sequester defensive compounds from host plants and potentially use them in chemical defense against bird predation [31]. The compound was identified as 3-caffeoyl-muco-quinic acid. Host plants of *E*. *marcellus* have been restricted to the genus *Asimina* [32]. This stimulant was present in pawpaw leaves, but concentrations were strongly dependent on the season, with gradual increases from spring through summer, then decreases by late summer as plants began to senesce. Note that two flavonoid glycosides, rutin (quercetin 3-*O*-rutinoside) and nicotiflorin (kaempferol 3-*O*-rutinoside), were found in the same leaves and appeared to function as oviposition deterrents. The authors noted that *E*. *marcellus* adult females avoided ovipositing on leaves with high concentrations of the two compounds [30]. Thus, the interaction between rutin, nicotiflorin, and 3-caffeoyl-muco-quinic acid negatively influenced oviposition behavior of *E*. *marcellus* females.

Although quercetin 3-glycoside, quercetin 3-rutinoside (rutin), and quercetin 3-rutinoside-7-glucoside were discovered in the American pawpaw tree, only quercetin 3-glucoside (isoquercetin) was sequestered by zebra swallowtail *E*. *marcellus* larvae [33]. The author also indicated that quercetin and/or kaempferol derivatives have been identified in 39% of papilionid species.

The Asian swallowtail butterfly *Papilio xuthus* L. is distributed primarily in Asia. Adult females selected host plants for oviposition using gustatory receptors on chemosensilla on their forelegs [34]. Females oviposited on leaves of the host plant *Citrus unshiu* Marc. (Family Rutaceae) in the presence of flavonoid glycosides (rutin, hesperidin, narirutin, and vicenin-2) and other compounds such as adenosine, and 5-hydroxy-Nω-methyl-tryptamine [35]. An artificial blend of these compounds elicited an oviposition response comparable to that observed via *P*. *xuthus* tarsal contact with the leaf surface.

The zeryntiine swallowtail butterfly *Luehdorfia japonica* Leech (Family Papilionidae) oviposits on host plants in the genus *Heterotropa* (Family Aristolochiaceae). This butterfly is distributed throughout the Japanese archipelago [36]. Leaves of a host plant *Heterotropa* (*Asarum*) *asperum* F. Maek (Japanese wild ginger) contained one component of an oviposition stimulant, i.e., isorhamnetin 3-*O*-glucosyl-(1→6)-galactoside-7-*O*-glucoside [37]. This flavonoid glycoside alone did not elicit an oviposition response in adult females. Combining it with other unidentified compounds in a mixture elicited a specific oviposition response by *L*. *japonica*.

Females of the spangle butterfly *Papilio protenor demetrius* Cramer (Family Papilionidae) detected chemical cues on the surface of host plant foliage via chemoreceptors on the fifth foretarsi of their forelegs [38]. A subsequent study reported that *P*. *protenor* females were stimulated to oviposit in the presence of two flavonoid glycosides from the host plant *Citrus natsudaidai* (Yu. Tanaka) Hayata (Rutaceae) in Japan [39]. These included naringin, found in the epicarp and leaves, and hesperidin, found in the peels of *C*. *natsudaidai*. These two compounds had to be present in a mixture with other unidentified compounds before oviposition stimulation occurred in *P*. *protenor* females [39]. A follow-up investigation determined that a mixture of L-(-)-stachydrine, D-(-)-quinic, (-)-synephrine, and L-(-)-proline acted synergistically with naringin and hesperidin to stimulate *P*. *protenor* oviposition [40]. The flavonoid glycoside neohesperidin found in leaves and peel of *C*. *natsudaidai* did not stimulate *P*. *protenor* oviposition [40].

The flavonoid glycoside phellamurin was isolated from foliage of the Amur cork tree *Phellodendron amurense* Rupr. (Family Rutaceae) and did not stimulate oviposition in *P*. *protenor* and *Papilio xuthus* L., to a lesser extent [41]. The two species were sympatric, occupying similar host ranges. The authors suggested that the high concentration of phellamurin in *P*. *amurense* foliage reduced competition for oviposition sites between the two *Papilio* species. Although *P*. *protenor* and *P*. *xuthus* larvae consumed *P*. *amurense* foliage, only one species, that is *P*. *xuthus* oviposited on *P*. *amurense* foliage, regardless of the high concentration of phellamurin in foliage.

Oviposition by the spangle butterfly *P*. *protenor* and the alpine black swallowtail *Papilio maackii* Ménétries was affected by phellamurin [42]. Both species developed adequately on *P*. *amurense* foliage in the laboratory, but only the *P*. *maackii* oviposited on this plant under natural field conditions. The authors tested whether the presence of phellamurin in *P*. *amurense* foliage inhibited oviposition in *P*. *protenor*. Results indicated that phellamurin stimulated oviposition by *P*. *maackii* at concentrations ranging from 0.2% (20 μg/cm^2^) to 2.0% (200 μg/cm^2^) applied to plastic leaf surrogates in the laboratory. At 2% phellamurin, 72% of *P*. *maackii* females oviposited in the laboratory. Notably, 2% phellamurin was approximately the quantity found in young *P*. *amurense* leaves in the field. In contrast, phellamurin had negative effects on oviposition in *P*. *protenor*, even at a low concentration of 0.1% (10 μg/cm^2^) in the laboratory [42].

The common blue butterfly *Polyommatus icarus* (Rottemburg) (Family Lycaenidae) is distributed in diverse habitats throughout the Palearctic region [43]. Research was conducted to obtain knowledge on the sequestration and distribution of flavonoid compounds in *P*. *icarus* [44,45]. The authors [44] reared larvae on inflorescences of crownvetch *S*. *varia* and alfalfa *M*. *sativa*. They discovered that *P*. *icarus* larvae obtained flavonoid compounds when feeding on foliage of these host plants. Based on an analysis of larval feces, *P*. *icarus* sequestered (stored) primarily astragalin (kaempferol 3-*O*-glucoside) in their bodies. This compound was also the predominant flavonoid found in pupae and adults. Markedly, *P*. *icarus* adults stored this compound predominantly as color pigments in the wings rather than in body tissues. Larvae were also reared on inflorescences of white clover *Trifolium repens* L. (Family Fabaceae) in the laboratory [45]. Hyperoside (quercetin 3-*O*-galactoside) was the main compound sequestered from *T*. *repens* by *P*. *icarus* larvae. The authors also indicated that larvae metabolized flavonoids via feeding on *T*. *repens* inflorescences. As such, flavonoid content, including a kaempferol derivative not found in *T*. *repens* inflorescences, tended to increase from mature larvae, to pupae, to emerged adults. Interestingly, adult males and especially females accumulated flavonoids in their wings, in the form of color pigments. Since flavonoid content was two-fold greater in wings of females than males, color pigments could have served as visual cues to attract males to females to initiate mating behavior [45]. These results suggest that sequestered (stored) compounds (astragalin, and hyperoside) have a positive effect on mating behavior of *P*. *icarus*.

The flavonoid content was greater in *P*. *icarus* larval, pupal, and adult stages when individuals were reared on natural rather than unnatural host plants [46]. Flavonoids were primarily distributed in wings of males and females, but decidedly more concentrated in females. Additionally, larger-sized females had greater flavonoid loads. The authors suggested that flavonoids (color pigments in wings) can absorb UV light, indicative of a protective function. The higher UV-absorbing flavonoids in females might attract males to females to entice mating behavior [46].

The flavonoid content of adult *P*. *icarus* reared on *Vicia villosa* Roth (Family Fabaceae), an unnatural host plant, was determined in the laboratory [47]. Three out of four flavonoid glycosides were identified in *V*. *villosa* inflorescences. These included myricitrin (myricetin 3-*O*-rhamnoside), quercitrin (quercetin 3-*O*-rhamnoside), and afzelin (kaempferol 3-*O*-rhamnoside). Larvae consumed *V*. *villosa* leaves and metabolized (and presumably sequestered) quercitrin and afzelin. Afzelin was predominant in adults, representing 55% of the total flavonoid content. This compound was mostly incorporated into the wing patterns of adult *P*. *icarus*, females more than males. The flavonoids incorporated into wings could function in mate selection and recognition, with males being attracted to females [47]. In field experiments, *P*. *icarus* males were attracted to female dummies (models), which were artificially treated with flavonoids [48]. Males were more attracted to female models with high rather than low flavonoid content. These results suggest that quercitrin and afzelin have positive effects on *P*. *icarus* mating behavior.

The Adonis blue butterfly *Lysandra* (*Polyommatus*) *bellargus* (Rottemburg) (Family Lycaenidae) is throughout the Palearctic region, but possibly declining in some areas, such as in Great Britain [49]. Larvae sequestered and metabolized flavonoids via consumption of leaves of its host plant *Securigera* (*Coronilla*) *varia* (L.) Lassen [50]. Two of the main flavonoids in the host plant were isovitexin and isoorientin. Interestingly, the isovitexin derivative, isoviten-2”-*O*-xyloside was the dominant compound identified in pupae and imagines of *L*. *bellargus*. Additionally, quercetin-*O*-glycosides and kaempferol-*O*-glycosides were minor components in pupae and imagines. The authors indicated that 80% of the flavonoid content was found in the wings of adults. Females contained significantly more flavonoid content than males [50].

Four quercetin and 16 kaempferol derivatives were present in the leaves of Tronchuda cabbage *Brassica oleraceae* L. var. *costata* DC [51]. Two other compounds readily identified in Tronchuda cabbage leaves, namely kaempferol 3-*O*-sophoroside-7-*O*-sophoroside and kaempferol 3-*O*-sophoroside-7-*O*-glucoside, were also present in larvae of the large white cabbage butterfly *Pieris brassicae* L. (Family Pieridae). This species is distributed in the Palearctic region and has the potential to become a pest of native species of Brassicaceae in some locations, e.g., New Zealand [52]. Although kaempferol 3-*O*-sophoroside was a minor flavonoid derivative in foliage, it was one of the dominant flavonoids identified in *P*. *brassicae* larvae. Interestingly, quercetin derivatives were present in trace amounts in Tronchuda cabbage but in high amounts in *P*. *brassicae* larvae, suggesting that larvae sequestered and metabolized these dietary flavonoids, with no apparent negative effects on their development [51].

A total of 18 flavonoids, including derivatives, were discovered in wings and, to a lesser extent, in the body, including reproductive tissues of adult females, of the marbled white butterfly *Melanargia galathea* (L.) (Family Nymphalidae), collected from various host plant grasses (Gramineae, species not mentioned) in the field [53]. Briefly stated, flavonoids ingested by larvae (while feeding on host grass species) included isovitexin, vitexin, iso-orientin, orientin, luteolin, apigenin, tricin, and their glucoside derivatives. The author demonstrated that these flavonoids, and their derivatives, were present in *M*. *galathea* egg, larval, pupal, and adult life stages, suggesting that these compounds were sequestered and/or metabolized by *M*. *galathea*. The authors did not indicate whether the sequestered flavonoids had any effect on *M*. *galathea* behavior or life history. In a subsequent investigation, the flavonoid profiles discovered in *M*. *galathea* were related to the flavonoid content present in larval host plants [54]. Additionally, *M*. *galathea* larvae reared on the same grass species had matching flavonoid profiles [54].

Wing coloration was strongly related to flavonoid content in *M*. *galathea* adults in four populations in the United Kingdom [55]. Close examination of the wings revealed three main colors, i.e., yellow, cream, and white. Flavonoid content was 19.6 μg of flavone/mg of wing tissue for yellow-colored wings, 14.1 μg/mg for cream-colored wings, and 8.3 μg/mg for white-colored wings. Additionally, flavonoids constituted approximately 1.9%, 1.4%, and 0.8% of the weight of wing tissue of yellow-, cream-, and white-colored wings, respectively. As indicated in previous works by the author, flavonoid content was higher in the wings of *M*. *galathea* females than males.

The flavonoid content in body and wings of the Chalk-hill blue butterfly *Polyommatus* (*Lysandra*) *coridon* (Poda) (Family Lycaenidae) and 15 related species was highly dependent on the host plant fed upon by larvae [56]. Note that *P*. *coridon* has several subspecies and is distributed in the Palearctic region [57,58]. Examined host plants included *Lotus corniculatus* L., *Anthyllis vulneraria* L., and *Hippocrepis comosa* L., in the family Fabaceae. The flavonoids were kaempferol and four derivatives, quercetin and two derivatives, and two isorhamnetin derivatives. The author stated that the flavonoids were probably metabolized by *P*. *coridon* larvae, or by associated gut microflora, prior to sequestration. Although host plants contained flavones, isoflavones, and glycoflavones, only flavonols (i.e., kaempferol and quercetin) were sequestered by *P*. *coridon* larvae in this study [56]. The author did not mention whether sequestered compounds (kaempferol or quercetin) affected *P*. *coridon* behavior or life history.

In summary, five flavonoids were associated with butterfly behavior and life history (Table S3). The flavonoids included apigenin (one neutral effect), kaempferol (one neutral effect), luteolin (one neutral effect), quercetin (one neutral, one positive, and two negative effects), and tricin (one neutral effect). Irrespective of flavonoid compound, 62.5% (n = 5), 12.5% (n = 1), and 25% (n = 2) of the effects were neutral, positive, and negative, respectively. All neutral effects involved sequestration of flavonoid compounds; negative effects involved decreases in enzyme activity, and the one positive effect was an increase in enzyme activity (Table S3). A total of 18 flavonoid glycosides were associated with butterflies (Table S4). Four compounds demonstrating more than one effect included hesperidin (three positive), naringin (two positive), phellamurin (one positive and two negative), and rutin (one positive, one negative) (Table S4). Combining the effects from all flavonoid glycosides, 58.33% (n = 14), 25% (n = 6), and 16.66% (n = 4) of the effects were positive, neutral, and negative, respectively. There were a total of 24 effects. Sequestration of compounds from host plants represented four neutral effects and four positive effects.

### 2.2. Bees (Order Hymneoptera)

#### 2.2.1. Honey Bees

The western honey bee *A*. *mellifera* (Family Apidae, genus *Apis*) is transported worldwide and provides invaluable pollination of agricultural crops and produces honey, beeswax, and royal jelly [59,60]. Evidence of the antioxidant and antimicrobial properties of honey bee products has been accumulating in the literature [61,62]. Antimicrobial properties of honey could be strongly correlated with the presence of flavonoid molecules [63]. Quercetin was found in pollen and honey; honey bees fed on a diet of honey (which they generated from processed nectar) and bee bread ( a mixture of pollen and honey), from a diversity of plant species [64]. Honey bees recognized quercetin as a xenobiotic (foreign substance). According to the authors, quercetin was a substrate for the CYP6AS1, CYP6AS3, CYP6AS4, and CYP6AS10 subfamily of genes, belonging to the cytochrome P450 monooxygenases family (P450s), and the CYP6AS subfamily was important in the metabolism of quercetin. Although quercetin is a xenobiotic, it has a positive effect on enzyme production.

In a related study, Mao et al. [65] showed that the CYP9Q1, CYP9Q2, and CYP9Q3 genes, also of the P450 family, metabolized the acaricide tau-fluvalinate, which has been used to control the parasitic mite *Varroa destructor* Anderson and Trueman in *A*. *mellifera* hives. These three genes also detoxified the organophosphate coumaphos. Consumption of honey by *A*. *mellifera* worker bees could supply quercetin into the gut and induce CYP9Q2 and CYP9Q3 gene activity. The upregulation of quercetin could explain, at least in part, the ability of *A*. *mellifera* workers to detoxify these pesticides [65]. Similarly, p-coumaric acid (a phenolic acid), which is a precursor to the flavonoids naringenin and pinocembrin, is common in honey, pollen, and bee bread. It has upregulated P450s, particularly CYP9Q3 in *A*. *mellifera* [66]. Feeding *A*. *mellifera* adult workers a diet of sucrose combined with honey, propolis, or pollen upregulated CYP6AS genes, which encoded enzymes that metabolized quercetin and other flavonoids [67,68]. In addition, p-coumaric acid and the flavonoids pinocembrin and pinobanksin 5-methyl ether had positive benefits on *A*. *mellifera* by inducing CYP9Q genes, which encoded enzymes that metabolized quercetin and several acaricides [66,68].

As stated in previous work, quercetin in honey and pollen can be metabolized by P450s in the guts of *A*. *mellifera* workers. Quercetin was a xenobiotic and potentially harmful to *A*. *mellifera* at high concentrations. Additionally, the inefficient metabolism of quercetin decreased energy production (ATP) in *A*. *mellifera* [69]. For example, laboratory experiments revealed that ingestion of the fungicide triazole myclobutanil and the phytochemical quercetin resulted in less thoracic ATP, which is necessary for adequate functionality of flight muscles. Therefore, myclobutanil interfered with quercetin metabolism, which compromised energy production and reduced flight capacity in *A*. *mellifera* workers [69]. In this case, quercetin had a negative effect on *A*. *mellifera*.

Behavioral responses of *A*. *mellifera* workers to nine phytochemicals (including five flavonoids), frequently found in food sources, i.e., nectar, propolis, and pollen, and five pesticides (fungicides and herbicides), found to contaminate hives, were tested in the laboratory [70]. Of the flavonoids, quercetin at all concentrations (0.01, 0.05, 0.10, 0.25, and 0.50 mM), elicited more *A*. *mellifera* visitations to feeder cups (a measure of flight capacity) and greater sugar water consumption (feeding) rates than the other flavonoids. The other flavonoids included chrysin, galangin, naringenin, and pinocembrin. Chrysin (at 0.1 ppm) and pinocembrin (at 1 ppm) reduced *A*. *mellifera* visitations (flight) but did not affect sugar water consumption (feeding). However, galangin did not affect visitations (flight) or consumption (feeding). Naringenin (at 100 ppm) increased sugar water consumption (feeding) only. Of the pesticides, the herbicide glyphosate and fungicide chlorothalonil elicited *A*. *mellifera* behavioral responses. Workers preferred to consume glyphosate-sugar water at a concentration of 10 ppb versus a sugar water control. In contrast, workers preferred to consume chlorothalonil at 0.5 and 50 ppb; they preferred visiting at 0.5 ppb [70].

In a complementary study, researchers [71] determined the effects of quercetin and p-coumaric acid in the presence/absence of pyrethroids (4 ppm bifenthrin and 0.5 ppm β-cyfluthrin) on the lifespan of 1-day-old *A*. *mellifera* workers. When fed sugar syrup laced with the compounds, quercetin (hazard ratio, HR = 0.82) extended the lifespan of the workers. Both 4 ppm bifenthrin (HR = 9.17) and 0.5 ppm β-cyfluthrin (HR = 1.34) reduced the lifespan. The authors indicated that dietary quercetin increased the ability of the workers to tolerate both pyrethroids [71]. Therefore, quercetin had positive effects on *A*. *mellifera* lifespan.

Researchers [72] tested the capacity of quercetin and p-coumaric acid in a sugar-based diet, at a range of concentrations, to increase *A*. *mellifera* worker lifespan by reducing pesticide toxicity in laboratory bioassays. Pesticides included the fungicide propiconazole and the insecticide chlorantraniliprole; both were typically tank-mixed and sprayed onto almond trees during bloom in the central valley of California. At low to moderate concentrations, both quercetin (12.5–250.0 μM) and p-coumaric acid (5.0–50.0 μM), fed separately or in combination in sugar diets, tended to reduce the negative effects of both pesticides on *A*. *mellifera* worker lifespan [72].

The effects of feeding *A*. *mellifera* adults on a liquid diet spiked with quercetin and/or the fungicide boscalid on flight capacity and energy production were determined in an indoor flight treadmill [73]. Boscalid is a broad-spectrum fungicide that suppresses fungal diseases, such as white mold *Sclerotinia sclerotiorum* (Lib.) de Bary, grey mold *Botrytis cinerea* Pers., and powdery mildew *Uncinula necator* (Schwein.) Burrill during the bloom season in orchards. This compound inhibited mitochondrial respiration in pathogenic fungi but potentially interfered with mitochondrial respiration in *A*. *mellifera* workers foraging for honey and pollen during bloom season. In comparison to a solvent control, quercetin alone had positive effects on *A*. *mellifera* by increasing wingbeat frequency and adenosine triphosphate (ATP) production in flight muscles. Boscalid alone decreased *A*. *mellifera* wingbeat frequency. The combination of a diet of quercetin plus boscalid did not significantly affect wingbeat frequency, in comparison to the solvent control.

The potential interaction between the insecticide imidacloprid and quercetin was examined by another team of scientists [74]. Exposure and ingestion of low concentrations (15 and 45 ppb) of imidacloprid with quercetin improved the survival rate of *A*. *mellifera* workers. At much higher imidacloprid concentrations (105 and 135 ppb), dietary quercetin had the opposite effect of reducing *A*. *mellifera* survival rate. Therefore, quercetin had the positive effect of restoring the survival rate of *A*. *mellifera* workers after exposure to low concentrations of imidacloprid [74].

The inclusion of kaempferol in sucrose-based diets had a positive effect on *A*. *mellifera* survival in the face of infection by the pathogenic fungus *Nosema ceranae* (Fries) (Dissociodihaplophasida: Nosematidae) [75]. This fungus infested *A*. *mellifera* colonies and was responsible for colony collapses in some regions of the world [76]. Using eight-day-old *A*. *mellifera* workers in experimental cages, diets containing high (2500 ppm) rather than low (250 ppm, or 25 ppm) kaempferol concentrations versus control (sucrose only) extended the longevity of infected workers in one season. In the following season, there were no significant differences amongst concentrations (2500, 250, and 25 ppm), but all concentrations promoted greater worker survival versus control. Additionally, *N*. *ceranae* spore loads were lower in infected workers fed kaempferol, at all concentrations, than the control [75].

When mixed in sucrose-based diets, a mixture of one flavonoid, three flavonoid glycosides, and six phenolic acids reduced the negative effects of the nicotinoid thiacloprid on *A*. *mellifera* workers in cage bioassays [77]. The flavonoid was quercetin; the flavonoid glycosides were rutin, naringin, and hesperidin. The authors evaluated the worker mortality rate, daily food intake, and expression of P450s after exposure to thiacloprid in diets. Mortality rates were lower when workers were fed diets spiked with low (35 mg/L) and high (70 mg/L) thiacloprid concentration along with phenols rather than without phenols. In addition, daily food intake was greatest when workers were fed diets with phenols alone rather than with low or high thiacloprid concentrations. Because all phenolics were mixed and tested together, the contributions of the flavonoid and flavonoid glycosides to *A*. *mellifera* health could not be evaluated separately in this study.

*Apis mellifera* workers effectively pollinated bulb onion (*Allium cepa* L.) plants as they foraged for nectar [78]. To determine the influence of nectar compounds on the appetitive behavior of workers, experiments were set up near beehives. Workers of unknown age were restrained in wooden cages or metal tubes and subjected to sucrose-based diets spiked with potassium and naringenin, quercetin, or luteolin [79]. The authors tested two common onion lines, the MS (male sterile) line and the OP (fertile “open pollinated”) line, to determine if foraging workers responded behaviorally to potassium, naringenin, and quercetin in the nectar of flowers of the MS line, or to potassium and luteolin in the nectar of flowers of the OP line. Using artificial nectar mimics, the authors measured proboscis extension (after antennal contact) and food uptake behaviors of workers in response to the presence of these flavonoids. They discovered that potassium (3000 ppm) reduced proboscis extension and food uptake in the MS and OP nectar mimic treatments. Potassium (3000 ppm) and naringenin (10 ppm) and/or quercetin (1 ppm) decreased proboscis extension in the MS nectar mimic treatment. In contrast, potassium (3000 ppm) and luteolin (10 ppm) increased learning in the OP nectar mimic treatment. The authors indicated that proboscis extension and food uptake behaviors were estimates of *A*. *mellifera* learning capacity and nectar palatability, respectively [79].

The consumption of high-quality pollen by *A*. *mellifera* decreased the susceptibility to pesticide toxicity [80,81]. Kaempferol and quercetin glycosides were common in pollen [82]. One study evaluated the acute and chronic effects of sulfoxaflor (insecticide) and azoxystrobin (fungicide) on honey bees fed a pollen diet of variable quality [19]. Consumption of a high-quality (S) rather than a low-quality (BQ) pollen diet significantly reduced *A*. *mellifera* mortality resulting from chronic exposure to sulfoxaflor in the laboratory. Chronic exposure to azoxystrobin (0.2 to 2.0 ppm) did not cause mortality in workers, regardless of whether they were fed the low- or high-quality pollen diets. The S diet contained 89% *Salix* (willow) pollen; the BQ diet contained 36% Brassicaceae pollen and 35% *Quercus robur* L. (pedunculate oak) pollen, respectively. Quercetin concentration in diets was not detectable using the techniques employed in this study. Moreover, the importance of quercetin or kaempferol in reducing *A*. *mellifera* worker mortality after consuming the high-quality pollen was not clearly ascertained in this study [19]. 

*Apis mellifera* workers fed artificial nectar, i.e., 30% (w/w) sucrose syrup solution spiked with a high concentration (0.01%) rather than a low concentration (0.005%) of quercetin vs. a control, tended to resist “signals” from queen bees [83]. For example, workers fed nectar with high quercetin generated significantly more queen cells (compared to the low- and no-quercetin treatments). Additionally, some workers attacked their queens. However, ovarian development was significantly greater in workers fed nectar with high quercetin. The authors surmised that a high quercetin concentration in artificial nectar had a negative effect on *A*. *mellifera* colonies by interrupting the pheromonal communication system between workers and queens [83].

In laboratory experiments, quercetin increased *A*. *mellifera* worker sensitivity to the neonicotinoid insecticide acetamiprid [84]. For example, sucrose syrup diets containing quercetin (1 mM) and acetamiprid (0.1 μg/μL) caused a dramatic decrease in worker survival in comparison to the control, acetamiprid (0.1 μg/μL) alone. The authors mentioned that inadvertent combinations of secondary metabolites (e.g., quercetin) and pesticides can have negative effects on *A*. *mellifera* worker survival in hives.

Sucrose diets containing quercetin, rather than those without quercetin, reduced the concentration of the insecticide imidacloprid in the tissues of *A*. *mellifera* workers as determined by a novel QuEChERS method [85]. This observation suggests that quercetin has the potential to detoxify imidacloprid. However, feeding workers sucrose diets spiked with quercetin did not affect the concentrations of the fungicide tebuconazole and the insecticide/acaricide tau-fluvalinate in the body tissues of sacrificed individuals [85]. In a related investigation, researchers [86] examined pesticide (tebuconazole, tau-fluvalinate, and imidacloprid) concentrations in the body tissues of *A*. *mellifera* workers fed diets containing nectar and pollen from four plants including *Reseda odorata* L., *Borago officinalis* L., *Phacelia tanacetifolia* Benth., and *Trifolium repens* L., for 48 h. They discovered that these plants had different flavonoid profiles, contributing to varied concentrations of pesticide residues in worker tissues. Additionally, flavonoids tended to reduce residual concentrations of tau-fluvalinate and imidacloprid in worker tissues. Dietary pollen and nectar from *R*. *odorata* increased the metabolism of tebuconazole in tissues in *A*. *mellifera* workers. The authors suggested that plants rich in flavonoids (present in pollen and nectar) held promise in ameliorating the toxic effects of some pesticides on *A*. *mellifera* health [86]. In this study, flavonoid effects on *A*. *mellifera* worker health were not tested individually.

The Asian honey bee *Apis cerana* F. is the native domesticated (and wild) honey bee in Southern and Eastern Asia [87]. It produces smaller colonies than the western honey bee (*A*. *mellifera*). Importing *A*. *mellifera* into Asia could have led to declines in population densities and health of *A*. *cerana* [88]. In contrast, a recent study suggests that climate factors have been a key factor in *A*. *cerana* population declines in China [89]. Exposing *A*. *cerana* worker bees to a sublethal dose of imidacloprid (20 μg/L) reduced their responsiveness to sucrose only; exposure to a lethal dose (100 μg/L) reduced sucrose responsiveness and reduced longevity by 10 days, in comparison to the control [90]. However, feeding workers a low (37.8 mg/L) rather than a moderate to high (75.6 mg/L) concentration of quercetin for 24 h, prior to contact with imidacloprid, tended to restore longevity. This observation indicated that diets containing low concentrations of quercetin had the positive effect of detoxifying imidacloprid within the digestive tract of *A*. *cerana* workers.

#### 2.2.2. Bumble Bees

Bumble bees (Family Apidae, genus *Bombus*), such as *Bombus terrestris* (L.) and *Bombus impatiens* Cresson are important pollinators of crop plants in Europe and North America, respectively [91]. Because of their larger body size and ability to fly in cool temperatures, *Bombus* species are more effective pollinators than smaller-sized *Apis* species, especially in protected culture, i.e., glasshouses or greenhouses [92,93,94].

The literature on flavonoid-bumble bee interactions is very scant. In one study, the authors [95] noted that *B*. *terrestris* workers typically required a visual stimulus to initiate their orientation to flowers for the collection of pollen. Workers were attracted to artificial pollen, i.e., yellow-colored quercetin (quercetin dihydrate) pigments, as often as to natural pollen from the common dandelion *Taraxacum officinale* (L.) in laboratory bioassays. This suggests that quercetin had a neutral effect as a visual attractant for *A*. *mellifera*. In another study, the flavonoid glycoside rutin, which was present in pollen and nectar of many plant species, reduced harmful effects of a neurotoxic insecticide (fipronil) on cognitive (memory) retention in *B*. *impatiens* workers [96]. When used as a prophylactic in test diets, rutin caused workers to retain their cognitive abilities after the oral administration of sublethal doses of fipronil. The authors suggested that rutin could be researched more extensively for its positive effects in protecting *B*. *impatiens* workers from pesticide toxicity [96].

In summary, there were seven flavonoids associated with bee behavior and life history (Table S3). The flavonoids included chrysin (one neutral and one negative), galangin (two neutral), kaempferol (one positive), naringenin (one positive and one neutral), pinobanksin (two positive), pinocembrin (three positive, and one neutral), and quercetin (13 positive, two neutral, and two negative). When combining all flavonoids, 66.67% (n = 20), 23.33% (n = 7), and 10.0% (n = 3) of the effects were positive, neutral, or negative, respectively. The positive effects of flavonoid exposure often involved the detoxification of pesticide residues or a decrease in pesticide residue concentration in bees (Table S3). Other positive effects, neutral effects, and negative effects are listed in Table S3. Only one flavonoid glycoside, i.e., rutin, was associated with bee life history (Table S4). Rutin had one positive effect (and no neutral or negative effects). It functioned to increase the detoxification of pesticide residues by worker bees [96].

### 2.3. Sawflies (Order Hymenoptera)

Phytophagous sawflies (Order Hymenoptera: Family Diprionidae) have come into contact with flavonoids through their host plants. Some diprionid species in the subfamily Tenthredininae feed on perennial herbs and sequester iridoid glycosides [97] and flavonoid glycosides [98] from their host plants into various body parts. *Tenthredo zonula* Klug (subfamily Tenthredininae) larvae sequestered flavonoid glycosides from the foliage of *Hypericum perforatum* L. and *Hypericum hirsutum* L. (Family Hypericaceae). The compounds included rutin, hyperoside, quercitrin, and isoquercitrin. They were sequestered into the salivary glands, integument, and hemolymph, but not the digestive tract. Moreover, *T*. *zonula* larvae might store quercitrin in the hemolymph [98]. The authors suggested, with caution, that sequestered compounds served a defensive role, such as deterring ant predation [98]. Therefore, sequestration of flavonoid glycosides could have a positive (rather than neutral) effect on sawfly life history.

Research on managing sawfly populations has targeted species that defoliate conifers, e.g., pine (Family Pinaceae) trees [99]. The performance of two pine sawfly species *Diprion pini* (L.) and *Neodiprion sertifer* (Geoffroy) (Family Diprionidae), which are pests of conifers in Eurasia [100], in response to the presence or absence of taxifolin 3′-O-β-D-glucopyranoside (taxifolin 3-glucoside) in pine needles was evaluated in the laboratory [101]. Both species fed on Scots pine (*Pinus sylvestris* L.) needles, and this study examined two *P*. *sylvestris* chemotypes, one containing 2–4% taxifolin 3-glucoside content and another containing 0% taxifolin 3-glucoside content, on the larval performance of both sawfly species. Chemotypes were present within the same *P*. *sylvestris* populations in regions of Europe [102,103]. Results indicated that taxifolin 3-glucoside had no effect on body size or survival of female *N*. *sertifer* larvae and a minor negative effect on the development of female *D*. *pini* larvae. Development time was extended by 6% when larvae fed on needles of the chemotype containing 2–4% taxifolin 3-glucoside rather than the chemotype containing 0% taxifolin 3-glucoside. Males were not tested [101].

The performance of *D*. *pini* larvae, measured as a change in cocoon mass, was compared when they were subjected to feeding on *P*. *sylvestris* needles from previously defoliated versus undefoliated trees [104]. The authors noticed an accumulation of flavonoids and flavonoid glycosides in previously defoliated rather than undefoliated trees and correlated the concentration of these compounds with *D*. *pini* performance. The results indicated that defoliation induced a strong increase in the concentration of myricetin 3-galactoside, which correlated with a decrease in *D*. *pini* performance (cocoon mass). Additionally, (+)-catechin showed an increase in concentration, but weaker than that of myricetin 3-galactoside, which correlated with a decrease in *D*. *pini* performance (cocoon mass). Two other compounds, hyperoside (hyperin, quercetin 3-*O*-galactoside) and quercitrin, showed an increase in concentration with defoliation but did not correlate with *D*. *pini* performance [104].

The hemolymph and feces of fourth and fifth instar *N*. *sertifer* larvae fed *P*. *sylvestris* needles contained four flavonoid glycosides: (+)-catechin 7-*O*-β-glucoside, isorhamnetin 3,7,4′-tri-*O*-β-glucoside, kaempferol 3,7,4′-tri-*O*-β-glucoside, and quercetin 3,7,4′-tri-*O*-β-glucoside [105]. The concentration of these compounds, combined, was 3.7 mg/mL in the hemolymph. Interestingly, these compounds were not detected in the needles of the host plant. The authors suggested that *N*. *sertifer* larvae produced these flavonoid glycosides de novo from precursors, i.e., (+)-catechin and unidentified flavonoid glycosides, found inside *P*. *sylvestris* needles. The compounds were apparently stored in the larval hemolymph and might be involved in larval metabolism rather than defense against predators [105].

Researchers studied the biochemical modification of flavonoid compounds, identified in mountain birch *Betula pubescens* Ehrhart subsp. *czerepanovii* (N. I. Orlova) (Fagales: Betulaceae) mature leaves, in the digestive tract of sawfly species in Finland [106]. Note that mountain birch was native to northern Eurasia [107]. Sawfly species examined in this study included five tenthredinid species: *Nematus viridis* Stephens, *Nematus brevivalvis* Thomson, *Priophorus pallipes* Lepeletier, *Pristiphora alpestris* (Konow), *Amauronematus amplus* Konow, and one unidentified argid *Arge* sp. Shrank (Family Argidae) [106]. Purportedly, none of these species were of economic importance to mountain birch anywhere in northern Europe. Adults emerged from cocoons in summer, then females oviposited on mature leaves because young leaves were not usually available. Consequently, larvae were exposed to mature rather than young leaves due to phenological incongruence between leaf age and larval development. In the laboratory, authors [106] discovered that eight flavonoid glycosides present in the larval leaf diet were detected in the feces of sawfly species, discriminately. Five of these, i.e., quercetin 3-*O*-glucuronoside (miquelianin), quercetin 3-*O*-galactoside (hyperoside), myricetin 3-*O*-glucuronoside, myricetin 3-*O*-galactoside, and kaempferol 3-*O*-glucoside (astragalin), were found in the leaf diet and feces of all seven sawfly species [106]. The predominant compound was quercetin 3-*O*-glucuronoside in the leaf diet (ranging from 6.4–7.3 mg/g diet, dry wt) and in larval feces (ranging from 9.2–10.3 mg/g feces, dry wt). In contrast, acacetin 7-*O*-glucoside was not detected in the larval diet but was present in larval feces (0.5–0.7 mg/g feces, dry wt) of all sawfly species, suggesting that all species produced this compound from one or more birch leaf flavonoid glycosides [106].

In a follow-up investigation, researchers [108] “painted” flavonoid aglycones onto the surface of mountain birch leaves and offered them to larvae of six sawfly species, which differed in their seasonal phenology, i.e., early-, mid-to-late-, or late-season species. The six species were mentioned in the previous study by the same authors [106]. Results revealed that “painting” three flavonoids, i.e., kaempferide, acacetin, and naringenin 4′, 7-dimethylether, at a 10 mg/g concentration, onto the surface of leaves had a negative effect on the development of late-season and mid-to-late-season sawfly species. The experimental flavonoids had little or no effect on the early season species, although young (rather than mature) mountain birch leaves typically contained the highest flavonoid content. These observations suggested that early-season sawfly species, such as *A*. *amplus* and *P*. *alpestris*, were well-adapted to feeding on mountain birch leaves with high flavonoid content, with little or no negative consequences to growth and development [108].

Researchers [109] tested the hypothesis that flavonoid mono-glycosides in birch foliage were used by sawfly larvae to produce flavonoid oligo-glycosides (i.e., tri-, tetra-, penta-, and hexa-glycosides), not found in the foliage of host plants. Nine sawfly species were fed diets containing foliage from three birch species, namely, *Betula pendula* Roth (silver birch), *Betula nana* L. (dwarf birch), and *B*. *pubescens* subspecies *czerepanovii* (mountain birch), which differed in the composition and content of foliar flavonoids [109]. The nine species of sawfly tested in this study included *A*. *amplus*, *P*. *alpestris*, *Dineura pullior* Schmidt and Walter (Family Tenthredinidae), *Trichiosoma scalesii* Leach (Family Cimbicidae), *Nematus pravus* (Konow) (Family Tenthredinidae), *N*. *viridis*, *N*. *viridescens*, *N*. *brevivalvis*, and *Arge* sp. The results indicated that flavonoid oligo-glycosides were produced by sawfly larvae reared on different diets and the content and concentration of these compounds differed amongst sawfly species. *Nematus pravus*, *N*. *viridis*, and *A*. *amplus* contained the highest total flavonoid content (approximately 11 mg/mL, mean value), whereas *Arge* sp., *P*. *alpestris*, and *N*. *brevivalvis* contained the lowest total flavonoid content (approximately 1.5 mg/mL, mean value). Note that kaempferol oligo-glycosides and quercetin oligo-glycosides were detected in larval hemolymph of all species, more in some species than others. The specific names of these compounds were not stated in this study. The effects of these compounds on sawfly life history were not reported. The authors concluded that the mechanism by which sawfly larvae produced new compounds, apparently de novo, was unknown.

In summary, there were just three flavonoids associated with sawfly life history (Table S3). The flavonoids included acacetin (one negative effect), (+)-catechin (one negative effect), and kaempferide (one negative effect). All three flavonoids (100%, n = 3) functioned negatively, decreasing larval development or decreasing cocoon mass (body weight) of sawflies. There were 13 flavonoid glycosides; they caused positive, neutral, and negative effects (Table S4). The compounds with more than one documented effect included hyperoside (one positive and one neutral), kaempferol 3,7,4′-triglucoside (two neutral), quercitrin (one neutral, and one positive), and taxifolin 3-glucoside (two neutral and one negative). See the other compounds in Table S4. Compiling the effects from the 13 flavonoid glycosides, the percentage of positive, neutral, and negative effects was 22.22% (n = 4), 66.67% (n = 12), and 11.11% (n = 2), respectively.

### 2.4. Beetles (Order Coleoptera)

#### 2.4.1. Pests

Many beetle species are pests of plants in forested and non-forested ecosystems throughout the world. Researchers [110] discovered D-catechin ((+)-catechin) within an acetone-based extract of the inner bark of Japanese red pine *Pinus densiflora* Siebold and Zucc. (Family Pinaceae). Experiments were conducted to determine if this compound could stimulate oviposition behavior of the Japanese pine sawyer *Monochamus alternatus* Hope (Family Cerambycidae). This cerambycid is a vector of the pine wood nematode *Bursaphelenchus xylophilus* (Steiner and Buhrer) Nickle, which is distributed in Asia, Europe, and North America, and the combination of the cerambycid and nematode causes mortality in pines [111]. Results indicated that D-catechin, plus a fraction of the extract applied to filter paper, stimulated oviposition behavior of *M*. *alternatus* females in laboratory bioassays. The active compound in the fraction was not identified [110].

Research discovered that the flavanone-3-hydroxylase enzyme in Norway spruce *Picea abies* (L.) H. Karst. (Family Pinaceae) was responsible for the production of phenolic compounds involved in host plant defense against the European spruce bark beetle *Ips typographus* (L.) (Family Curculionidae, subfamily Scolytinae) and its associated blue-stain fungus *Endoconidiophora polonica* (Siemaszko) (Family Ceratocystidaceae) [112]. The enzyme catalyzed the conversion of eriodictyol (a flavanone) to taxifolin, which is a precursor of catechin. The authors indicated that simulated fungal and beetle attack on Norway spruce increased the production of taxifolin and catechin. Laboratory bioassays using artificial media for *E*. *polonica* and an artificial diet for *I*. *typographus,* amended with these flavonoids, demonstrated negative effects on both pests [112]. Taxifolin and catechin (both at 1 mg/mL) reduced *I*. *typographus* feeding and tunneling behavior. Taxifolin and catechin (both at 2 mg/mL) reduced *E*. *polonica* growth and proliferation versus untreated controls. Taxifolin was more toxic than catechin to *E*. *polonica*. In a related investigation, *E*. *polonica* used Norway spruce defensive polyphenols, including flavonoids, e.g., catechin, as a food (carbon) source [113].

In this related study, authors evaluated the comparative ability of five species of fungi associated with the bark beetle *I*. *typographus* to degrade the phenolic-based defenses of Norway spruce [114]. Two other bark beetle-associated fungi, *Grosmannia penicillata* (Grosmann) and *Grosmannia europhioides* (E.F. Wright and Cain), degraded phenolic compounds more rapidly than *E*. *polonica* on artificial media. The degraded phenolics included catechin, gallocatechin, taxifolin glucoside, and quercetin glucoside. The authors noted that *I*. *typographus* adults showed a slight preference for tunneling into artificial medium containing fungi rather than phenolics. This observation suggested that fungi associated with *I*. *typographus* function in degrading host plant defenses, i.e., phenolics, thus creating a more favorable habitat for the exploitation of host resources (e.g., food) by *I*. *typographus*.

Feeding stimulants for adults of the smaller European elm bark beetle *Scolytus multistriatus* (Marsham) (Family Curculionidae, subfamily Scolytinae) were isolated from extracts of twig bark of American elm *Ulmus americana* L. (Family Ulmaceae) in Ohio, USA [115]. The compounds were identified as (+)-catechin-5-β-D-xylopyranoside and lupeyl cerotate. The combined compounds elicited an adult feeding response of 60–70% of that achieved with using the initial extract [115]. Note that the beetle *S*. *multistriatus* was the primary vector of Dutch elm disease, which caused significant mortality in elm trees in North America and Europe [116]. In a follow-up study, researchers [117] revised the configuration of the flavonoid component of the feeding stimulant for *S*. *multistriatus*. The revised formula was (+)-catechin-7-β-D-xylopyranoside (catechin 7-xyloside). Therefore, the feeding stimulant consisted of a combination of this flavonoid and lupeyl cerotate.

The boll weevil *Anthonomus grandis* Boheman (Family Curculionidae) has been a notorious pest of agricultural plants such as upland cotton *Gossypium hirsutum* L. (Family Malvaceae) in cotton-growing regions throughout the world. In recent years, integrated pest management strategies, involving pheromones, insecticidal sprays, or genetically modified (transgenic) *G*. *hirsutum* cultivars have eradicated *A*. *grandis* infestations in much of its distribution, although there has been evidence of its migration back into some eradication zones in North and Central America [118,119,120]. Laboratory experiments tested the utility of tannin, gossypol, rutin, and quercetin, occurring naturally in *G*. *hirsutum* flowers and buds, to affect feeding, development, and oviposition behavior of *A*. *grandis* [121]. Results indicated that neither rutin nor quercetin (at concentrations ranging from 0.10% to 1.0%) in larval diets had any effect on feeding or oviposition. However, quercetin at a 0.6% concentration in larval diets caused an increase in mean body mass (in mg) of emerged *A*. *grandis* adults when compared to the control. Rutin (at the concentrations tested) did not significantly influence the mean body mass of emerged adults when compared to the control [121]. These results suggest that quercetin, at a low concentration, has the positive effect of increasing the body mass of *A*. *grandis* adults.

A related study determined the potential of flavonoids and other compounds isolated from *G*. *hirsutum* flower buds to stimulate feeding behavior of *A*. *grandis* adults [122]. The authors determined that quercetin, quercetin-3′-glucoside, and quercetin 7-glucoside were capable of stimulating feeding behavior. All compounds were tested at a concentration of 100 μg in bioassays in the laboratory.

Researchers [123] studied the host plant resistance mechanisms in two duckweed species, *Lemna perpusilla* Torr. and *Lemna minor* L. (Family Araceae), which were free-floating aquatic plants, in response to herbivory from the duckweed weevil *Tanysphyrus lemnae* Paykull (Family Curculionidae). They discovered that flavone glucosides isovitexin, vitexin, and isoorientin were found in the fronds of both duckweed species but were more prevalent in *L*. *minor* than *L*. *perpusilla*, regardless of weevil herbivory. They also discovered that hesperetin, a flavanone glycoside, was more abundant in the fronds of *L*. *perpusilla* than in *L*. *minor*, regardless of weevil herbivory or not [123]. Moreover, *T*. *lemnae* females preferred to oviposit on *L*. *perpusilla* rather than on *L*. *minor*. Similarly, larval development and survival rates were greater on *L*. *perpusilla* than on *L*. *minor*. Apparently, isovitexin, vitexin, and isoorientin in *L*. *minor* deterred feeding and oviposition behavior of *T*. *lemnae* larvae and adult females, respectively.

Scientists [124] identified flavonoids in the adzuki bean *Vigna angularis* (Willd.) Ohwi & H. Ohashi (Family Fabaceae) in the laboratory, then determined if these compounds stimulated oviposition behavior in the adzuki bean weevil *Callosobruchus chinensis* (L.) (Family Chrysomelidae, subfamily Bruchinae). This chrysomelid has been a major pest of *Vigna* beans, but resistant varieties have been developed and natural enemies have been identified to curb infestations [125,126]. Results indicated that quercetin, taxifolin, and D-catechin ((+)-catechin) were discovered in an aqueous extract of *V*. *angularis* seed coat [124]. Taxifolin (concentration, 2.0 ng per glass bead) and D-catechin (concentration, 0.2–2.0 ng per glass bead) stimulated oviposition behavior, but quercetin did not [124]. In a previous investigation, D-catechin (concentration, 0.2–2.0 ng per glass bead) was also found to stimulate oviposition behavior in *C*. *chinensis* adults [127].

Authors tested the effects of partially purified flavonoids isolated from the Sodom apple plant *Calotropis procera* (Aiton) W.T.Aiton (Family Apocynaceae), and flavonoids and flavonoid glycosides from a commercial supplier, on the immature and adult stages of *C*. *chinensis* [128]. The compounds included quercetin, quercitrin, rutin, myricetin, fisetin, and an unidentified flavone. In filter paper diffusion bioassays, the *C*. *procera* partially purified flavonoids and quercetin, at a concentration of 10 mg/mL, caused 100% and 48% adult mortality, respectively, two days after contact. The other compounds caused little or no mortality at the same concentration, in comparison to untreated control. Treatment of stored grain (a food source) with *C*. *procera* partially purified flavonoids and quercetin at 10 mg/mL caused 40% and 29% adult mortality, respectively, after two days of exposure. The other compounds caused little or no mortality at the same concentration [128]. Moreover, the *C*. *procera* partially purified flavonoids and quercetin (at 10 mg/mL) had negative effects on *C*. *chinensis* oviposition, adult emergence, and body mass of emerged adults. Note that at the 5 mg/mL concentration, all compounds reduced *C*. *chinensis* body mass when compared to the untreated control.

Two non-flavonoids, salicin and populin, and one flavonoid glucoside, luteolin-7-glucoside, were identified in leaves of the deciduous shrubs *Salix gracilistyla* Miq. and *Salix gilgiana* Seemen (Family Salicaceae) [129]. Laboratory bioassays were conducted to determine the variation in feeding responses of several chrysomelids, which commonly defoliate several *Salix* species, when confronted with these compounds. The chrysomelids included *Chrysomela vigintipunctata* Scopoli, *Plagiodera versicolora* (Laicharting), and *Lochmaea capreae* (L.) adults. All species, especially *C*. *vigintipunctata* and *P*. *versicolora,* feed on foliage of *Salix* species in Europe [130]. Results of laboratory bioassays revealed that all chrysomelids were stimulated to feed, i.e., nibble, on filter paper treated with variable concentrations of luteolin-7-glucoside [129]. Both *C*. *vigintipunctata* and *L*. *capreae* responded favorably to 0.1 M (molar) and 0.01 M (molar) concentrations in a water-based or 0.1 M in a sucrose-based solution. On the other hand, *P*. *versicolora* responded to 0.1 M and 0.01 M concentrations in a 0.01 M sucrose-based solution of this compound. None of the chrysomelids responded to the 0.001 M concentration in water or 0.1 M sucrose-based solutions [129]. The authors indicated that none of the chrysomelids responded to the flavonoid luteolin at any test concentrations (0.001 to 0.1 M) in sucrose- or water-based solutions.

In a companion study, authors [131] tested the feeding stimulation effects of flavonoids and flavonoid glycosides on eight chrysomelid species. The compounds included morin, myricetin, myricitrin, quercetin, quercitrin, and rutin at a 0.01 M concentration in a 0.5 M sucrose solution, in comparison to the control (0.5 M sucrose solution). *Salix*-feeding *P*. *versicolora* adults were stimulated to feed, i.e., nibble, on filter paper (in Petri dish arenas) treated with all these compounds. Note that feeding stimulatory responses of *P*. *versicolora* were greatest for myricetin, myricitrin, and quercetin, but least for morin [131].

Glucosinolates in horseradish *Armoracia rusticana* G. Gaertn. (Family Brassicaceae) leaves stimulated feeding behavior of the flea beetle *Phyllotreta armoraciae* (Koch) (Family Chrysomelidae). Larvae sequestered glucosinolates from horseradish leaves and stored them in their hemolymph. These compounds may function in a defensive capacity against predators [132,133]. Researchers [134] conducted feeding bioassays to determine if a quercetin glycoside and a kaempferol glycoside, identified in horseradish leaves, contributed to the selection of this plant as the primary host of *P*. *armoraciae* in the field. Results indicated that kaempferol 3-*O*-xylosylgalactoside stimulated feeding, but quercetin 3-*O*-xylosylgalactoside did not [134]. These structures are not listed in Table S2, and effects are not listed in Table S4.

Crucifer flea beetle (*Phyllotreta cruciferae* (Goeze)) larvae and adults feed on leaves of canola (*Brassica napus* L. (Family Brassicaceae)) but not on leaves of the closely related false flax (*Camelina sativa* (L.)) [135]. Canola leaves contain large quantities of kaempferol glycosides, whereas false flax leaves contain large quantities of quercetin glycosides, with small quantities of rutin. The quercetin glycosides in false flax leaves deterred herbivory by *P*. *cruciferae*. Moreover, only trace quantities of quercetin glycosides were present in canola leaves [135]. The authors also demonstrated that taxifolin deterred feeding, whereas apigenin, hesperetin, isorhamnetin, and naringenin stimulated feeding in this beetle [135].

Taxifolin and quercetin increased the mortality of first instars of an insecticide-resistant strain of the Colorado potato beetle *Leptinotarsa decemlineata* Say (Family Chrysomelidae) in the laboratory [136]. Taxifolin inhibited the activity of glutathione S-transferases (GST) and functioned as a synergist in combination with insecticides such as Guthion (i.e., 50% azinphos-methyl). Insecticide-resistant and non-resistant strains of *L*. *decemlineata* were highly damaging defoliators of cultivated potato *Solanum tuberosum* L. (Family Solanaceae) in North America, Europe, and China [137].

Secondary metabolites in corn (maize) such as ferulic acid, rutin, and quercetin modified the feeding behavior of the dried-fruit beetle *Carpophilus hemipterus* (L.) (Family Nitidulidae) in the laboratory [138]. This nitidulid is distributed in North America, Europe, and Oceania. It has been an important pest of corn, stone fruit, and vegetables [139,140]. When incorporated into an artificial diet, ferulic acid (1000 ppm, dry wt) reduced feeding by *C*. *hemipterus* larvae and adults when compared against the control in no-choice bioassays. In contrast, incorporating rutin or quercetin (at 1000 ppm, dry wt) into an artificial diet increased *C*. *hemipterus* feeding behavior vs. control in choice bioassays [138].

Flavonoids isolated from extracts of apricot twig bark elicited attraction and biting (feeding) behavior in adults of the fruit tree bark beetle *Scolytus mediterraneus* Eggers (Family Curculionidae, subfamily Scolytinae) in laboratory bioassays involving the Styropor disk method [141]. This beetle is an important pest of deciduous fruit trees, especially apricot, in the Mediterranean region [142,143]. Of the six flavonoids identified, taxifolin, pinocembrin, and dihydrokaempferol (aromadendrin) provoked high feeding activity; kaempferol, quercetin, and naringenin provoked low feeding activity at test concentrations of 0.002, 0.02, and 0.2 mg/Styropor disk [141]. The authors concluded that at least three flavonoids, i.e., those provoking high activity, were involved in feeding stimulation. However, field tests are necessary to confirm the laboratory results.

In Nigeria, researchers [144] tested the effects of quercetin, which was isolated in a chloroform extract of bark from the stem of the snake bean plant *Bobgunnia madagascariensis* (Desv.) (Family Fabaceae), as an antifeedant for the red flour beetle *Tribolium castaneum* Herbst (Family Tenebrionidae), a serious pest of stored grain, e.g., maize. The wafer disc choice bioassay method was used to determine feeding responses to quercetin in the laboratory. Quercetin, at concentrations of 5 mg/mL and 10 mg/mL, deterred feeding activity of *T*. *castaneum* adults by 54.04% in bioassays. Azadirachtin deterred feeding activity by 67.10% [144]. Adults were not deterred from feeding on n-hexane extracts of the plant. Note that quercetin glycosides and kaempferol glycosides were found in the seed pods of *B*. *madagascariensis* [145].

Flavonoids represent a cadre of phenolic compounds involved in apple (*Malus* species, Family Rosaceae) host plant resistance against attack from herbivorous insects. Adults of the polyphagous and highly invasive Japanese beetle *Popillia japonica* Newman (Family Scarabaeidae) feed preferentially on Rosaceae foliage [146,147]. Scientists [148] conducted experiments to determine the feeding responses of *P*. *japonica* adults to rutin, quercetin, catechin, naringenin, and kaempferol, presented in artificial diets in laboratory bioassays, at concentrations ranging from 0 to 100 mM (millimolar). Results indicated that rutin and quercetin stimulated feeding at the higher concentrations, catechin and naringenin deterred feeding at the higher concentrations, and kaempferol did not elicit any feeding response regardless of concentration [148]. These results suggest that flavonoids have multifunctional effects on *P*. *japonica* feeding behavior under laboratory conditions.

In a similar investigation, the feeding behavior of *P*. *japonica* adult females was challenged by 22 natural compounds, including flavonoids and flavonoid glycosides, found in different cultivars of *Malus* species via incorporation into an artificial diet [149]. Rutin, quercetin, catechin, and naringenin were tested in the laboratory at concentrations ranging from 0 to 100 mM (millimolar). Results revealed that increasing the concentration of rutin in diets caused an increase in *P*. *japonica* feeding behavior. Increasing concentrations of quercetin and catechin, tested separately, caused an initial increase in feeding at lower concentrations, but decreased feeding at higher concentrations. Increasing the concentration of naringenin decreased *P*. *japonica* feeding behavior [149].

Research evaluated the effectiveness of rutin and quercetin, extracted from buckwheat *Fagopyrum esculentum* Moench (Family Polygonaceae), as antifeedants against the European cockchafer *Melolontha melolontha* L. (Family Scarabaeidae) [150]. This beetle is an important pest of grasses, small fruits, and tree fruit in European countries [151,152]. Buckwheat could be exploited as a natural source of rutin, quercetin, and other phytochemicals [153]. Neither rutin (20 mg/mL) nor quercetin (20 mg/mL) deterred grub feeding on 2-year-old Scots pine *Pinus sylvestris* L. (Family Pinaceae) seedling roots growing in soil medium in pots when compared against the control [150].

Flavonoids play a role in interactions amongst phytophagous lady beetles (Family Coccinellidae, subfamily Epilachninae) and their host plants. *Epilachna paenulata* Germar feed on the leaves of crop plants in the Family Cucurbitaceae in South America [154]. This species and others in the genus *Epilachna* produce defensive compounds (e.g., alkaloids) de novo in their bodies, and there is evidence that adults transfer some of these compounds to their offspring (egg stage) via mating [155,156]. Researchers [154] evaluated the effects of quercetin and pinocembrin on *E*. *paenulata* larval feeding behavior, development, and survival rate in laboratory bioassays. Experiments involved spraying cotyledons (seed leaves) of a host plant, cultivated squash *Cucurbita maxima* Duchesne, with different concentrations (0, 0.1, 1, 5, and 50 μg/cm^2^) of quercetin or pinocembrin and monitoring *E*. *paenulata* responses. Results indicated that quercetin had little to no negative effects on *E*. *paenulata* consumption rate or larval body weight at the tested concentrations (0, 0.1, 1, 5, and 50 μg/cm^2^). Larval survival only slightly decreased at higher concentrations of quercetin (5 and 50 μg/cm^2^). In contrast, pinocembrin (at 1, 5, 50 μg/cm^2^) reduced consumption rate and deterred any feeding after nine days at higher concentrations (5, 50 μg/cm^2^). Moreover, pinocembrin (at 1, 5, 50 μg/cm^2^) reduced body weight and survival rate of *E*. *paenulata* larvae [154]. The authors concluded that pinocembrin was a feeding deterrent, but quercetin was not.

#### 2.4.2. Predators

The pink-spotted lady beetle *Coleomegilla maculata* (DeGeer) (Family Coccinellidae, subfamily Coccinellinae) is an important predator of aphids and other soft-bodied arthropods found in North, Central, and South America [157,158]. Laboratory experiments evaluated the effect of flavonoids on oviposition (egg clutch production) by *C*. *maculata* [159,160]. Taxifolin, quercetin, and naringenin, identified in fractionated extracts of heartwood sawdust of Eastern red cedar *Juniperus virginiana* L. (Order Cupressales: Family Cupressaceae), stimulated oviposition (egg clutch production) by *C*. *maculata* females in laboratory micro-cages [159]. Moreover, these compounds altered the oviposition site preference of females. They oviposited at the base of micro-cages, adjacent to the compounds, rather than on the sidewalls and lid, which were the normal oviposition sites. In a follow-up investigation, experiments involved using quercetin from a commercial supplier. The results were consistent; commercial quercetin increased egg clutch production 1.5-fold (versus control) and altered oviposition site preference by *C*. *maculata* [160].

The variegated lady beetle *Hippodamia variegata* (Goeze) (Family Coccinellidae, subfamily Coccinellinae) is an important predator of aphids and other soft-bodied insects. It was first discovered in the Palearctic region but has been introduced, intentionally or unintentionally, to the Nearctic and Oriental regions [161,162]. Research evaluated the effects of two cultivars of cucumber (*Cucumis sativus* L., Family Cucurbitaceae), Storm and Kasib, on the interactions between *H*. *variegata*, its prey, i.e., melon aphid, *Aphis gossypii* Glover (Order Hemiptera: Family Aphididae), reared on Storm or Kasib cultivars [163]. The total flavonoid (quercetin standard) content in cucumber leaves was higher in the Storm than in the Kasib cultivar. Moreover, the Storm cultivar had a positive effect on the health of the predator *H*. *variegata*. For instance, *H*. *variegata* larval stages developed faster, and adult females produced more progeny when fed *A*. *gossypii* nymphs reared on leaves of the *C*. *sativus* Storm cultivar rather than the Kasib cultivar [163].

In summary, there were 14 flavonoids associated with beetle life history (Table S3). The flavonoids had positive, negative, or neutral effects on beetles. The flavonoids expressing more than one effect included catechin (four positive and three negative), fisetin (one negative and one neutral), kaempferol (one negative and one neutral), myricetin (one positive and one negative), naringenin (one positive and four negative), pinocembrin (one positive and three negative), quercetin (seven positive, nine negative, and six neutral), and taxifolin (three positive and three negative). Combining all 14 flavonoids, there were 58 total effects on beetles. The percentage of positive, neutral, and negative effects was 37.93% (n = 22), 18.96% (n = 11), and 43.10% (n = 25), respectively.

The 12 flavonoid glycosides had positive, negative, and neutral effects on beetles (Table S4). The compounds found to have more than one effect included isoorientin (two negative), isovitexin (two negative), quercitrin (one negative, one positive, and one neutral), rutin (one negative, four positive, and five neutral), and vitexin (two negative). Combining all flavonoid glycosides, there were 26 total effects on beetles. The percentage of positive, neutral, and negative effects was 38.46% (n = 10), 30.77% (n = 8), and 30.77% (n = 8), respectively.

### 2.5. True Bugs (Order Heteroptera)

#### 2.5.1. Predators

The zoophytophagous bug *Orius sauteri* (Poppius) (Family: Anthocoridae) is a predator of small-bodied insects, including aphids. It also imbibes small quantities of nutrients from plant foliage [164]. The authors tested the effects of tomato *Solanum lycopersicum* L. (Family: Solanaceae) cultivars expressing high flavonoid content (NIL-PH) vs. low flavonoid content (NIL-GH) on life history parameters of *O*. *sauteri* in the laboratory. The specific flavonoid compounds in the two cultivars were not mentioned in this article. However, plants with high flavonoid content were resistant to attack by whiteflies *Bemisia tabaci* (Gannadius) (Family: Aleyrodidae). Even in the presence of prey (whiteflies), *O*. *sauteri* oviposition, development time, and survival were affected negatively when inhabiting plants with high (rather than low) flavonoid content. In conclusion, tomato plants that express high flavonoid levels disrupt the biological control potential of *O*. *sauteri* [164]. A companion study by the authors elaborated on the role of tomato plants expressing high flavonoid content as a valid form of host plant resistance against attacks by *B*. *tabaci* [165].

Another investigation tested the indirect effects of rutin on the growth and development of the predatory bug *Podisus maculiventris* (Say) (Family Pentatomidae) in the laboratory [166]. Prey, i.e., tobacco hornworm *Manduca sexta* (L.) (Order Lepidoptera: Family Sphingidae) larvae were fed an artificial diet containing different concentrations of rutin (0, 6, 12, or 18 μmoles per gram of diet, fresh wt). When consuming prey that fed on the diet containing the highest rutin concentration, fifth instar *P*. *maculiventris* experienced a decline in relative growth rate, but this effect was evident at a warm (28 °C) rather than a cold (18 °C) temperature. Diet effects were more pronounced on females than on males [166].

#### 2.5.2. Pests

Flavonoids have negative effects on the health of pest bugs such as aphids (Family Aphididae), which attack crop plants in agroecosystems throughout the world [20]. Examples of these effects are described in the current and subsequent paragraphs. Screening of cultivated lines of cowpea *Vigna unguiculata* L. Walp (Family Fabaceae) for their flavonoid content revealed that they all contained quercetin, kaempferol, and isorhamnetin [167]. The aphid-resistant *V*. *unguiculata* lines (rather than the susceptible lines) had a higher flavonoid content. In bioassays in the laboratory, quercetin and isorhamnetin were effective at a 0.1 mM concentration in inhibiting production of progeny by the black bean aphid *Aphis fabae* (Scopoli) by a rate of 50%. Kaempferol was least effective (less than 20%) in inhibiting *A*. *fabae* progeny production.

The potential of quercetin and naringenin to disrupt development, fecundity, and longevity (survival) of the pea aphid *Acyrthosiphon pisum* Harris was determined in the laboratory using an artificial diet rather than live host plants [168]. Probing into the artificial diet for *A*. *pisum* females was also investigated in this study. Results indicated that both flavonoids had detrimental effects on *A*. *pisum* development, fecundity, and longevity at higher concentrations of 1000 and 10,000 μg/cm^3^. However, probing behavior (i.e., number of stylet penetrations) was significantly greater when adult females were exposed to quercetin in a liquid artificial diet at 10, 100, and 1000 μg/cm^3^ versus control. Naringenin did not alter the number of penetrations at any concentration in comparison to the control [168].

Female *A*. *pisum* adult penetration of and feeding on alfalfa *Medicago sativa* L. (Family Fabaceae) foliage correlated negatively with apigenin glycoside content; *A*. *pisum* abundance decreased as apigenin glycoside content increased [169]. Six apigenin glycosides were identified in *M*. *sativa* foliage. The total content of the six apigenin glycosides, combined, in infested and non-infested *M*. *sativa* foliage was 3.99 and 3.55 mg/g dry wt of foliage, respectively [169]. In a closely related study, researchers [170] discovered that luteolin, tricin, and chrysoeriol glycosides were dominant in the flavonoid profiles of *A*. *pisum* infested and non-infested *M*. *sativa* foliage. The authors did not detect a significant difference in flavonoid glycoside content between *A*. *pisum* infested and un-infested *M*. *sativa* foliage. The authors noted that tricin glycoside concentration was positively correlated with aphid abundance on foliage. Moreover, luteolin glycoside and chrysoeriol glycoside content was positively correlated with aphid fecundity per female. However, tricin, luteolin, or chrysoeriol glycoside concentration in foliage did not affect aphid feeding behavior [170].

Scientists tested the effects of genistein and luteolin, incorporated into separate artificial diets, on the feeding behavior of adult *A*. *pisum* females [171]. Test concentrations in the diets ranged from 0, 10, 100, to 1000 μg/cm^3^. Both compounds prolonged the time of *A*. *pisum* stylet probing into the diet, reduced the production of saliva, and passive ingestion of the diet. Moreover, production of saliva was completely halted in *A*. *pisum* at high concentrations (1000 μg/cm^3^ for genistein and 100 μg/cm^3^ for luteolin). The authors suggested that this knowledge could be used to develop plants with increased resistance to aphid herbivory [171].

The effects of genistein on the populations of two host races of the pea aphid *A*. *pisum* were examined in the laboratory [172]. The host races included *A*. *pisum* adapted to feeding on alfalfa *M*. *sativa* (*Medicago* host race) and the other one adapted to feeding on pea *Pisum sativum* L. (*Pisum* host race). When incorporated into an artificial diet, genistein had hardly any effects on the survival of second instar nymphs of the *Medicago* host race. In contrast, genistein significantly reduced the survival of second instar nymphs of the *Pisum* host race, as concentration increased from 0, 1, to 10 μg/mL [172].

The probing behavior of *A*. *pisum* was challenged by kaempferol, daidzein, apigenin, and genistein [173]. Methanol-based solutions of these flavonoids were prepared, and pea *P*. *sativum* leaves were then dipped into the solutions. After 8 h, leaves dipped in 0.1% kaempferol, daidzein, and apigenin solutions decreased the ingestion of pea leaf sap versus control. Genistein did not affect sap ingestion by *A*. *pisum*. Using the electrical penetration graph technique, the probing behavior, i.e., stylet penetration of leaf tissue, of *A*. *pisum*, *Myzus persicae* (Sulzer), and *Rhopalosiphum padi* (L.) was evaluated after exposure to quercetin and rutin [174]. The peach-potato aphid *M*. *persicae* has vectored more than 100 plant viruses among plants classified within over 40 families [175]. Results indicated that quercetin increased probing of leaf tissue by *A*. *pisum* and *M*. *persicae*. In contrast, rutin delayed tissue penetration by *A*. *pisum* and deterred probing behavior altogether by *M*. *persicae*. There was a clear concentration effect of quercetin and rutin on probing by *A*. *pisum* and *M*. *persicae*; intensity of behavioral responses increased as concentration increased from 0%, 0.1%, and 0.5% [174].

Researchers evaluated the flavonoid content in water-stressed and aphid-infested thale cress *Arabidopsis thaliana* (L.) (Family Brassicaceae) leaves in the laboratory [176]. Two aphid species, *M*. *persicae* and *Brevicoryne brassicae* (L.) were reared on *Brassica rapa* ssp. *chinensis* (Family Brassicaceae) foliage in an insectarium. In the experiment, both aphid species were subjected to water-stressed treatments that included well-watered (control), drought, and waterlogged conditions. Results indicated that kaempferol, quercetin, and isorhamnetin content (μmol/gm, dry wt) of waterlogged plants, without aphid feeding, increased significantly. Aphid feeding (especially by *M*. *persicae*) decreased flavonoid content of drought stressed and waterlogged plants. Overall, kaempferol was slightly more abundant than quercetin and isorhamnetin in *A*. *thaliana* [176]. The decrease in flavonoid content in stressed plants could have represented a defensive response to aphid herbivory.

*Sorghum bicolor* (L.) Moench plants have been important crops for food, fiber, and bioenergy production throughout the world. The corn leaf aphid *Rhopalosiphum maidis* Fitch was one of the major aphid species attacking sorghum. Host plant resistance in *S*. *bicolor* in the form of 3-deoxyflavonoids (3-DFs) and 3-deoxyanthocyanidins (3-DAs) was evaluated against *R*. *maidis* in cage experiments and laboratory bioassays [177]. A MYB transcription factor, yellow seed1 (y1), regulates expression of 3-DFs and 3-DAs in *S*. *bicolor*. Therefore, functional y1 plants contain these compounds, but non-functional null y1 plants do not. As expected, *R*. *maidis* showed a distinct preference for feeding and reproducing on null y1 plants in cage experiments and laboratory bioassays. Moreover, *R*. *maidis* experienced higher mortality when reared on an artificial diet spiked with functional y1-regulated compounds rather than null y1-regulated flavonoids [177]. Therefore, 3-DFs and 3-DAs have toxic effects on *R*. *maidis*.

In another study, *S*. *bicolor* plants produced more flavonoids in leaf tissue in response to *R*. *maidis* herbivory [178]. Flavonoid content, i.e., rutin equivalents (RE), increased significantly as aphid density increased, in comparison to non-infested control plants, from medium density (200 aphids per plant) to high density (300 aphids per plant). The authors also indicated that leaf carotenoid content was reduced by 40% at the high aphid density.

Laboratory bioassays were set up to evaluate the utilization of rutin hydrate, quercetin dihydrate, and naringin (a naringenin glycoside) as alternative aphicides against the woolly apple aphid *Eriosoma lanigerum* (Hausmann) in Jordan, Middle East [179]. *Eriosoma lanigerum* is an important pest of apple *Malus domestica* Borkhausen [180]. Treatments involved two flavonoids and one flavonoid glycoside formulated in dimethyl sulfoxide (DMSO) at 0, 100, 1000, and 10,000 ppm concentrations. The study also included a non-treated negative control (distilled water plus DMSO) and a positive control (imidacloprid). All compounds caused mortality (reduced survival rate) of *E*. *lanigerum* nymphs more than adults, especially at the highest concentration, when compared with the negative control. After 24 h and 72 h of exposure to *M*. *domestica* shoots treated with 10,000 ppm, *E*. *lanigerum* nymphal mortality was 85–93% and 93–98%, respectively. The authors indicated that rutin hydrate was the most toxic of the three test compounds. Comparatively, after 24 h and 72 h of exposure to shoots treated with imidacloprid (at 10,000 ppm), nymphal mortality was 90% and 100%, respectively [179].

Researchers [179] also evaluated the effects of rutin hydrate, quercetin dihydrate, and naringin on a hymenopterous parasitoid *Aphelinus mali* (Hald.) (Order Hymenoptera: Family Aphelinidae), which was an important natural enemy of *E*. *lanigerum*. Treatments involved dipping *E*. *lanigerum* mummies (containing *A*. *mali* developing pupae) in the three test compounds and positive control (imidacloprid) at the highest concentration (10,000 ppm) only for approximately 10 s. Accordingly, 27, 31, and 35% of mummies treated with naringin, quercetin dihydrate, and rutin hydrate failed to yield *A*. *mali* adults. In contrast, 88% of mummies treated with 10,000 ppm imidacloprid did not yield any *A*. *mali* adults. Less than 10% of mummies treated with DMSO failed to yield *A*. *mali* adults. In comparison to imidacloprid, naringin, quercetin dihydrate, and rutin hydrate had minimal negative effects on *A*. *mali* emergence [179]. At a lower concentration, i.e., 1000 or 100 ppm, the three test compounds would probably not affect *A*. *mali* emergence.

Feeding behavior and life table parameters of the cotton/melon aphid *A*. *gossypii* were determined in response to two cucumber *Cucumis sativus* L. cultivars, i.e., Storm and Khasib, which differed in phenol content and trichome density [163]. This economically important aphid species is distributed in temperate and tropical regions around the world [181]. The leaves from plants of the Storm cultivar contained higher total flavonoid content, higher phenol content, less chlorophyll, and more trichomes in comparison to leaves from the Khasib cultivar. Total flavonoids in plant tissues of the two cultivars were calculated using a quercetin standard curve generated from a spectrophotometer [163]. Flavonoid content (quercetin equivalents/gram of un-infested leaf fresh weight) in Storm and Khasib leaves was 2.17 ± 0.07 mg and 1.49 ± 0.09 mg, respectively. Conversely, the flavonoid content in *A*. *gossypii* infested leaves of the Storm and Khasib cultivars was 3.11 ± 0.08 mg and 2.44 ± 0.09 mg, respectively. Moreover, *A*. *gossypii* nymphs developed more slowly, and adult females produced fewer offspring and survived fewer days when feeding on leaves of the Storm rather than the Khasib cultivar. According to life table parameters, *A*. *gossypii* intrinsic and finite rates of increase were lower when feeding on Storm rather than Khasib, population doubling time and mean generation time were slower on Storm than Khasib, and net and gross reproductive rates were lower on Storm than Khasib [163].

Researchers [182] examined the potential of the flavonoid epigallocatechin gallate (EGCG) as a repellent against two *A*. *gossypii* biotypes, i.e., the cotton biotype and cucurbit (cucumber) biotype. The results indicated that EGCG reduced the population density (survival rate) of *A*. *gossypii* at the biotype level. After five days of feeding on treated foliage, *A*. *gossypii* populations began declining rapidly. After 20 and 24 days, all individuals of the cotton and cucumber biotypes, respectively, had died [182].

Flavonoids and flavonoid glycosides found in common wheat *Triticum aestivum* L. (Family Poaceae) leaves affected aphid feeding behavior. Feeding rate of *Schizaphis graminum* (Rondani) and *M*. *persicae* decreased as concentrations of quercitrin, ranging from 0, 0.05, 0.10, 0.15, 0.20, and 0.25% (w/w) in a synthetic diet, increased in laboratory bioassays [183]. Moreover, in eight hours, the percentage of feeding declined to 30% at a concentration of 0.20% (w/w) quercitrin in the diet.

In summary, there were nine flavonoids associated with true bug life history (Table S3). Flavonoids with more than one effect included genistein (two negative and two neutral), kaempferol (one negative and one neutral), naringenin (three negative), and quercetin (six negative). There were 20 total effects. The percentage of positive, neutral, and negative effects was 0%, 15% (n = 3), and 85% (n = 17), respectively. There were three flavonoid glycosides; those with more than one effect included rutin (three negative). Combining all flavonoid glycosides resulted in five total effects; 100% (n = 5) of the effects were negative (Table S4).

### 2.6. True Flies (Order Diptera)

Research to discover eco-friendly, plant-derived natural products to control true flies of medical importance, such as mosquitoes (Family Culicidae) has been ongoing [184,185]. The flavonoids genistein and daidzein, isolated from *Streptomyces* isolate 85–88 culture medium supplemented with soybean meal, were toxic to yellow fever mosquito *Aedes aegypti* (L.) larvae in laboratory bioassays [186]. At 50 and 100 ppm, genistein caused 100% and 100% larval mortality, respectively, in 24 and 48 h; daidzein caused 90% and 100% larval mortality, respectively, in 24 and 48 h. The mode of action of genistein or daidzein was unknown [186].

The mode of action of daidzein as a larvicide against *A*. *aegypti* could involve inhibiting the activity of ecdysteroidogenic glutathione S-transferase Noppera-bo (Nobo) [187]. This enzyme is responsible for the synthesis of ecdysone in *A*. *aegypti*. Nobo inhibition correlated positively with the larvicidal activity of daidzein. Additionally, the flavonoid desmethylglycitein (DMG) was more effective than daidzein in Nobo inhibition. DMG was also a more effective larvicide. In a laboratory bioassay testing 0, 1, 10, and 100 ppm concentrations of daidzein versus DMG, 50% mortality of *A*. *aegypti* first instar larvae (i.e., LD50) was evident at 85.8 ppm and 9.39 ppm for daidzein and DMG, respectively. This indicated that DMG was more effective because it caused larval mortality at a lower dose. Moreover, at a sublethal dose (2.5 ppm), DMG inhibited larval growth as determined by measuring head capsule sizes in first instar *A*. *aegypti* larvae exposed to DMG versus control [187].

The flavonoids karanjin and karanjachromene, and the rotenoid flavonoid pongarotene, were isolated from seeds of the karanja tree *Millettia pinnata* (L.) (Order Fabales: Family Fabaceae) and tested as larvicides against *A*. *aegypti*, the northern house mosquito *Culex pipiens pallens* (Coquillett), and the Asian tiger mosquito *Aedes albopictus* (Skuse) in the laboratory [188]. Using liquid formulations of the test compounds and positive controls, i.e., temephos and fenthion, in direct contact bioassays against third instar larvae, the authors determined that karanjin was the most toxic flavonoid against all three species. The 24-h LC50 values for karanjin were 14.61 mg/L (*C*. *pipiens pallens*), 16.13 mg/L (*A*. *aegypti*), and 35.26 mg/L (*A*. *albopictus*). The 24-h LC50 values for karanjachromene were 18.74 mg/L (*C*. *pipiens pallens*), 20.57 mg/L (*A*. *aegypti*), and 52.97 mg/L (*A*. *albopictus*), indicating that this flavonoid was toxic to at least two species (*C*. *pipiens pallens*, and *A*. *aegypti*). The possible mode of action of karanjin and karanjachromene was by inhibiting acetylcholinesterase enzyme (AChE) activity in larvae [187]. Safety testing of karanjin or karanjachromene against non-target aquatic organisms, which consume mosquito larvae, will be required prior to widespread application into aquatic ecosystems.

Three flavonoid glycosides, i.e., rhoifolin, poncirin, and naringin, found in leaves of trifoliate orange *Poncirus trifoliata* (L.) (Order Sapindales: Family Rutaceae) had negative effects on *A*. *aegypti* immature and adult stages [189]. In 24-h bioassays against *A*. *aegypti* larvae, lethal concentration (LC90) values ranged from 0.15 to 0.22 mg/L. Similarly, all compounds demonstrated ovicidal activity. Female oviposition and egg hatch rates decreased as flavonoid concentration increased. At a concentration of 10 mg/L, these compounds repelled adult females from biting human subjects. Rhoifolin was most effective, followed by poncirin, then naringin [189].

The potential of using a quercetin nanosuspension (instead of quercetin in its natural form) as a biocide against *A*. *aegypti* larvae was tested in a laboratory investigation [190]. Of the range of concentrations tested (0, 100, 250, 375, and 500 ppm), 100 ppm and 500 ppm of the nanosuspension caused mortality of 44% and 100% of *A*. *aegypti* larvae in 48 h. Note that the quercetin nanosuspension was less toxic than quercetin (in its natural state) to a non-target aquatic microalgae *Chlorella vulgaris* Beijerink (Order Chlorellales: Family Chlorellaceae) at the higher concentrations (375 ppm and 500 ppm) in laboratory bioassays [190].

Flavonoids can also affect the health of true flies of agricultural importance. The fungus gnat *Lycoriella pleuroti* Yang et Zhang (Family Sciaridae), which feeds on edible fungi, e.g., oyster mushroom *Pleurotus ostreatus* (Jacq. ex Fr.) P. Kumm. (Family Pleurotaceae) in the larval stages, was tested against *Bt* (CryIAc) protein, gossypol, tannin, and quercetin in artificial culture media [191]. Gossypol and tannin had the greatest negative effects on *L*. *pleuroti*. Quercetin also had negative effects; percent mortality was 36.6% at 0.1% quercetin but just 5.2% at 0% quercetin in the culture medium. Although mortality declined to 28.8% and 27.6% at 0.2% and 0.3% quercetin concentrations, all quercetin concentrations (0.1, 0.2, and 0.3%) caused greater mortality than the control [191].

The vinegar or pomace fly *Drosophila melanogaster* Meigen (Family Drosophilidae) is a household pest of overripe or decaying fruit but is most widely known as a model organism in genetics research [192]. Researchers [193] tested the effects of quercetin on meiotic recombination and chromosome segregation in *D*. *melanogaster* females after being reared on a 5% quercetin diet versus control (artificial diet lacking quercetin). Note that the quercetin diet also contained glucose, corn meal, and yeast. The results indicated that quercetin did not alter chromosome segregation in comparison to the control. The same authors found that the quercetin diet had positive effects on *D*. *melanogaster* reproduction. In comparison to the control diet, adults reared on the quercetin diet yielded 10% more offspring over two consecutive generations [193].

The capacity of phenolics to function as biocides against the larvae of the apple maggot *Rhagoletis pomonella* (Walsh) (Family Tephritidae) was evaluated in the laboratory [194]. This species is a serious pest of apples in northeastern North America [195]. Phenols in several crab apple (*Malus* spp.) varieties demonstrated host plant resistance against *R*. *pomonella*. When incorporated into an artificial diet at a high concentration, i.e., 1000 ppm, D-catechin, naringenin, and quercetin inhibited *R*. *pomonella* egg hatch and larval development [194].

Scientists [196] tested the effects of quercetin in an artificial diet (at 0, 0.005, 0.05, 0.5, or 1.75% dry weight concentrations) on development of wild-type *D*. *melanogaster* larvae in the laboratory. Results indicated that the 1.75% quercetin diet had a positive effect on *D*. *melanogaster* development; larvae required less time to metamorphose into pupae. The other quercetin diet concentrations (and the control diet) did not affect development time. The authors also observed histological changes in appearance, i.e., coloration, of fat body cells in *D*. *melanogaster* larvae. Larvae (third instars) fed the 1.75% quercetin diet had dark-colored structures in some fat body cells. The dark-colored structures might have represented quercetin metabolites [196].

Another study tested the effects of quercetin on the growth and development of immature stages of the melon fruit fly *Bactrocera cucurbitae* (Coquillett) (Family Tephritidae) [197]. This tephritid is a significant pest of melons, cucumbers, tomatoes, and related crops in temperate, tropical, and subtropical areas of the world, especially in Hawaii, USA [198]. In laboratory trials, quercetin (at 0, 1, 5, 25, 125, 625, and 3125 ppm concentrations) affected *B*. *cucurbitae* immature growth, development, and adult emergence [197]. For instance, percentage egg hatch was significantly reduced after exposure to the highest quercetin concentration (3125 ppm) in comparison to the control (0 ppm); the other concentrations did not differ significantly from the control or the highest concentration. Larval body weight was significantly less than the control at 125, 625, and 3125 ppm quercetin concentrations versus control; pupal body weight was significantly less than the control only at the highest quercetin concentration (3125 ppm). The percentage emergence of *B*. *cucurbitae* adults was significantly reduced after 88–96-hour-old larvae had been treated with most quercetin concentrations (5, 25, 125, 625, and 3125 ppm) versus control [197]. In a follow-up study, researchers [199] found that quercetin had a negative effect on *B*. *cucurbitae* oviposition behavior. Egg laying and puncturing the substrate (2.5 cm^3^ pumpkin pieces) were reduced significantly when females were exposed to one quercetin concentration (125 ppm) versus control.

The effects of chrysin on growth, development, and oviposition behavior of the melon fruit fly *B*. (*Zeugodacus*) *cucurbitae* were determined in laboratory bioassays [200]. This flavonoid was formulated into a casein-based artificial diet (for larvae) at the following treatment concentrations: 5, 25, 125, 625, and 3125 ppm versus control (water). Results indicated that first instar larvae took approximately one day longer to complete development when fed the 3125 ppm chrysin diet treatment vs. control. Additionally, 34% and 73% of first instars on the 3125-ppm diet treatment and the control, respectively, reached the pupal stage. The percentage of adults emerging from pupae was 0% and 60% when first instars were fed the 3125-ppm diet treatment and the control, respectively. The number of oviposition punctures (ovipunctures) into air-dried pieces of pumpkin was significantly fewer for gravid females on the 25, 125, 625, and 3125 diet treatments vs. control. However, egg production per female was not significantly affected by any chrysin treatment vs. control in no-choice bioassays. The authors concluded that chrysin could be utilized in a pest management program against *B*. (*Zeugodacus*) *cucurbitae* [200].

In summary, there were nine flavonoids associated with the life history of true flies (Table S3). Flavonoids with more than two effects included catechin (two negative effects), chrysin (four negative and one neutral), daidzein (two negative), desmethylglycitein (three negative), naringenin (two negative), and quercetin (two positive, one neutral, and eight negative). There were 28 total effects. The percentage of positive, neutral, and negative effects was 7.14% (n = 2), 7.14% (n = 2), and 85.71% (n = 24), respectively. There were only three flavonoid glycosides associated with true flies. These included naringin (four negative), poncirin (four negative), and rhoifolin (four negative). As indicated, 100% (n = 12) of the effects were negative (Table S4).

## 3. Harmless or Harmful Outcomes of Flavonoids and Flavonoid Glycosides

As indicated in the previous section, flavonoids and flavonoid glycosides had positive, neutral, or negative effects on insect life history parameters (Tables S3 and S4). The positive and neutral effects can be categorized as harmless outcomes. The negative effects can be categorized as harmful outcomes. In Table 1, the proportion of harmless and harmful outcomes was compared within insect taxa, then analyzed statistically.

### 3.1. Outcomes on Beneficial Insects

When summarizing the data, irrespective of specific compounds and insect species, flavonoids caused significantly more harmless (than harmful) outcomes on bees. For example, harmless outcomes involved the use of flavonoids, e.g., quercetin, to reduce the deleterious effects of pesticide exposure to honey bees [74,77]. Quercetin was often present in many pollen species. The possibility of deploying artificial pollen (spiked with quercetin) as food for foraging and pollinating bees in crop fields under pesticide spraying regimes might be a novel strategy to preserve honey bees by boosting their tolerance to pesticide residues in advance. Quercetin was also found to have harmless outcomes on honey bees in a previous review that focused solely on quercetin [20]. The limited data on flavonoid glycoside interactions with bees prevented statistical analysis.

Although not significantly different, flavonoids showed some tendency to cause more harmless (than harmful) outcomes on butterflies (Table 1). Flavonoid glycosides caused significant harmless outcomes in butterflies. Common harmless outcomes included functioning as feeding and oviposition stimulants, mating attractants and stimulants, and chemical protectants against UV light in some butterfly species [28,46,48]. Another common harmless outcome was the sequestration of flavonoids from host plants. Although more research is needed, sequestration of compounds could play a role in fostering mating behavior in some butterfly species. This information could be useful in zoological gardens and museums where butterflies are mass-produced on natural host plants. The identification and isolation of harmless and biologically active flavonoids from host plants could streamline the rearing process by improving mating in captivity and reducing the need for massive amounts of natural host plants as food. The development of artificial diets spiked with certain flavonoids should be encouraged.

Although the data were limited, flavonoids had harmless outcomes on predatory beetles [159,160,163]. However, data on herbivorous and predatory species were not teased apart in the analysis in Table 1. Flavonoid glycosides have not been tested against predatory beetles. Unfortunately, flavonoids, at high concentrations, had harmful outcomes for predatory true bugs [164,166]. These observations signal an incompatibility between the deployment of predatory bugs for biological control and flavonoids as potential biocides against plant pests. However, this perceived incompatibility could be relaxed by carefully manipulating predator release rates and flavonoid concentrations (low, moderate, or high).

### 3.2. Outcomes on Pest Insects

Flavonoid glycosides caused significantly more harmless (than harmful) outcomes in sawflies (Table 1). In contrast, the data on flavonoid interactions with sawflies were too small for statistical analysis. Sequestration of flavonoid glycosides (and their derivatives) from host plant foliage was a common harmless outcome of exposure. There is also evidence that sequestration of compounds could play a defensive function in protecting sawflies from predation.

Flavonoids caused significantly more harmful (than harmless) outcomes on true bugs (Heteroptera). The data on flavonoid glycoside interactions were too limited for statistical analysis, but there was an apparent tendency for more harmful (than harmless) outcomes (Table 1). Flavonoids caused significantly more harmful (than harmless) outcomes in true flies (Diptera); flavonoid glycosides tended to produce the same outcomes (Table 1).

Flavonoids did not have significantly more harmful outcomes in beetles. Flavonoid glycosides almost had significantly more harmless outcomes in herbivorous beetles (Table 1). Nevertheless, the harmless outcomes suggest that some herbivorous species have adapted to flavonoid glycosides and use these compounds as chemical cues, in some instances, to stimulate feeding, oviposition, or to locate suitable host plants.

## 4. Utilization of Flavonoids and Flavonoid Glycosides in Pest Management

### 4.1. Sawflies

This study highlights the intimate connection and adaptation of sawfly species to their host plants. This information could provide clues for developing a flavonoid (or flavonoid glycoside) systemic insecticide that could be incorporated into plant tissues to deter sawfly larvae from feeding on tree foliage. Alternatively, breeding plant cultivars expressing high concentrations of flavonoids (acacetin, (+)-catechin, or kaempferide) or flavonoid glycosides (myricetin-3-galactoside) known to cause harmful outcomes in sawflies could be a useful management technique. Genetic modification of flavonoid-producing plants could help increase production of flavonoids for improved plant resistance against herbivory [133].

### 4.2. Beetles (Pests)

Some herbivorous beetles have adapted to feeding on host plants and could utilize flavonoids or flavonoid glycosides as chemical cues to locate suitable hosts. The potential of using these compounds as functional insecticides is a major challenge. Several flavonoids (quercetin, taxifolin, and pinocembrin) reduced the survival rates of a few beetle species in the laboratory [128,136,154]. Field tests are necessary to determine if these compounds can function as insecticides in the field. Most commonly, the negative effects were sublethal. For example, quercetin, naringenin, (+)-catechin, and pinocembrin, at high concentrations, reduced the feeding behavior of beetle species in the laboratory [148,149,154]. Perhaps these flavonoids could be formulated into an effective feeding deterrent. In contrast, other compounds, e.g., rutin, increased feeding behavior at high concentrations [148,149]. Perhaps rutin could function as a feeding stimulant in toxic baits.

### 4.3. True Bugs (Pests)

True bugs (Heteroptera) are likely targets for successful control by flavonoids due to the high proportion of harmful outcomes (see Table 1). This study revealed that flavonoids (genistein, quercetin, and naringenin) and flavonoid glycosides (rutin and naringin) reduced the survival rates of true bugs, but primarily at high concentrations [168,172,179]. This evidence suggests that these compounds could function as insecticides, but more realistically as repellents, under field conditions. These and other compounds had sublethal effects by reducing growth, development, feeding behavior, and reproduction, commonly at high concentrations in the laboratory (see Tables S3 and S4). Perhaps utilizing the compounds demonstrating sublethal effects as growth inhibitors, feeding deterrents, or oviposition deterrents would be realistic functions.

### 4.4. True Flies (Pests)

True flies are also likely targets for successful control by flavonoids due to a high proportion of harmful outcomes (see Table 1). Most notable is the study in which a nanosuspension of quercetin reduced the survival rate of mosquito larvae in the laboratory [190]. This suggests that quercetin could be an effective insecticide. Additionally, genistein and daidzein reduced the survival rates of mosquito larvae in the laboratory [186]. Developing formulations that persist in the field and in aquatic ecosystems has great potential for success [190]. The utilization of nanotechnology to improve sustainability of flavonoids under field application in aquatic as well as terrestrial ecosystems could be a viable strategy [201].

Several flavonoids (quercetin, DMG, karanjin, karanjachromene) and flavonoid glycosides (naringin, poncirin, rhoifolin) reduced the survival rates of terrestrial flies in the laboratory. This suggests that these compounds could function as insecticides in the field, but experiments are necessary to confirm this assertion.

## 5. Conclusions

This study has reviewed the literature on flavonoid and flavonoid glycoside interactions with insects. Key findings suggest that several compounds have moderate potential as insecticides or repellents against true bugs (Heteroptera) and true flies (Diptera). Several compounds have low potential as insecticides or repellents against sawflies and herbivorous beetles. Using flavonoids and flavonoid glycosides as feeding stimulants in toxic baits for beetle control is another possible use. Flavonoids and flavonoid glycosides are generally harmless to beneficial insects such as butterflies, bees, and predatory beetles.

A major concern with using flavonoids in pest management is the need to produce large quantities for field applications. Mass-producing flavonoids for broad-scale use in managed ecosystems could be a near-term endeavor [202,203,204,205]. The use of agricultural processing waste, e.g., citrus orange peels, as an abundant, readily available source of flavonoids could boost mass production [21,206,207]. Additionally, research to improve the persistence of these compounds in field applications is necessary.

## Figures and Tables

**Table 1:** A comparison of the proportion of harmless versus harmful outcomes in insects after exposure to flavonoids and flavonoid glycosides ^1^.

Taxa	Flavonoids		Statistics		Flavonoid Glycosides		Statistics	
	Harmless (*n*)	Harmful (*n)*	*Z*	*P*	Harmless (*n*)	Harmful (*n*)	*Z*	*P*
Butterflies	0.75 (6)	0.25 (2)	1.26	0.21	0.83 (20)	0.17 (4)	2.68	0.007
Bees	0.90 (27)	0.10 (3)	3.42	<0.001	1 (1)	0	==	==
Sawflies	0	1 (3)	==	==	0.89 (16)	0.11 (2)	2.61	0.009
Beetles	0.57 (33)	0.43 (25)	1.05	0.29	0.69 (18)	0.31 (8)	1.82	0.07
True Bugs	0.15 (3)	0.85 (17)	2.56	0.01	0	1 (5)	==	==
True Flies	0.14 (4)	0.86 (24)	3.03	0.002	0	1 (12)	==	==

^1^ Harmless outcomes represent positive or neutral effects on insects; harmful outcomes represent negative effects on insects (Tables S3 and S4). Sample size, *n*. Proportional effects in a row are significantly different if *p* < 0.05, *Z*-test. Double-dashed lines (==) indicate failure of the *Z*-test to perform an analysis due to zero values in the harmless outcome column.

## Data Availability

The data files supporting this research can be made available by the author on ResearchGate.

## Supplementary Materials

The following supporting information can be downloaded at: https://www.mdpi.com/article/10.3390/insects15120956/s1, Table S1: Flavonoids mentioned in this manuscript with common name, CAS number, IUPAC name, molecular weight, and chemical structure; Table S2: Flavonoid glycosides mentioned in this manuscript with common name, CAS number, IUPAC name, molecular weight, and chemical structure; Table S3: Flavonoids found to have positive (++), negative (--), or neutral (o) effects on insect behavior and life history parameters, with references; Table S4: Flavonoid glycosides found to have positive (++), negative (--), or neutral (o) effects on insect behavior and life history parameters, with references.
