# Supplementary material for: Evaluating the Effects of Flavonoids on Insects: Implications for Managing Pests Without Harming Beneficials
Source: Insects. 2024 Dec 1;15(12):956. doi: 10.3390/insects15120956 (PMC11678172; doi:10.3390/insects15120956)
Supplement: Supplementary file 1 [file insects-15-00956-s001.zip › insects-3255428-supplementary.pdf]

Supplementary Materials:

Table S1. Flavonoids mentioned in this manuscript with common name, CAS number, IUPAC name, molecular weight, and chemical structure. [1] .

Common name (CAS number)	IUPAC name	Molecular weight (g/mol)	Chemical structure
Acacetin (480-44-4)	5,7-dihydroxy-2-(4-methoxy-phenyl)chromen-4-one	284.26	$C_{16}H_{12}O_5$ 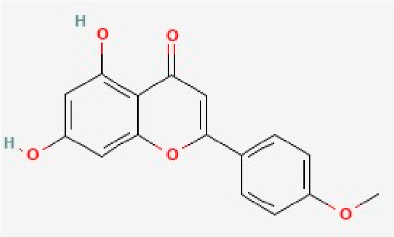
Apigenin (520-36-5)	4′,5,7-Trihydroxyflavone	270.24	$C_{15}H_{10}O_5$ 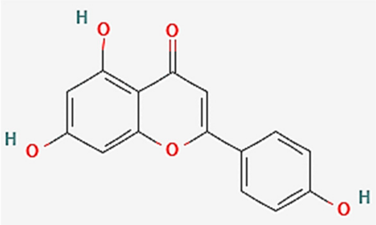
Aromadendrin (480-20-6) (Dihydrokaempferol)	(2*R*,3*R*)-3,4′,5,7-Tetrahy-droxyflavan-4-one	288.255	$C_{15}H_{12}O_6$ 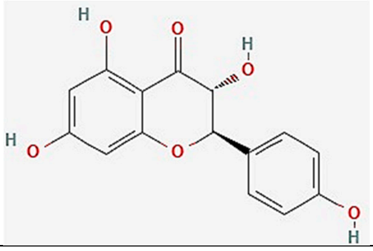
(+)-Catechin (7295-85-4)	(2*R*,3*S*)-2-(3,4-dihydroxy-phenyl)-3,4-dihydro-2*H*-chromene-3,5,7-triol	290.271	$C_{15}H_{14}O_6$ 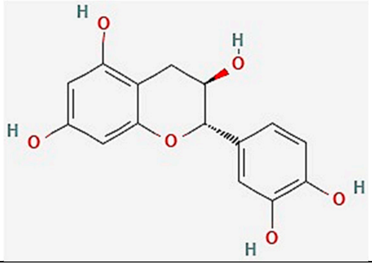
Chrysin (480-40-0)	5,7-Dihydroxyflavone	254.241	$C_{15}H_{10}O_4$ 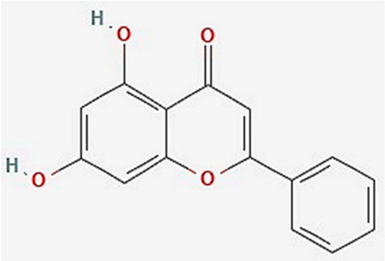

Daidzein<br>(486-66-8)	4′,7-Dihydroxyisoflavone	254.23	$C_{15}H_{10}O_4$<br>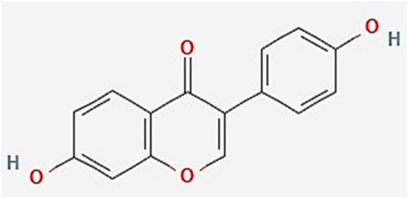
Desmethylglycitein (DMG)<br>(17817-31-1)<br>(4',6,7-Trihydroxyisoflavone)	6,7-Dihydroxy-3-(4-hydroxy-phenyl)chromen-4-one	270.24	$C_{15}H_{10}O_5$<br>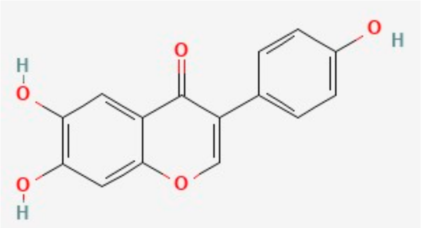
Epigallocatechin gallate<br>(EGCG)<br>(989-51-5)	[(2*R*,3*R*)-5,7-dihydroxy-2-(3,4,5-trihydroxyphenyl)-3,4-dihydro-2H-chromen-3-yl] 3,4,5-trihydroxybenzoate	458.4	$C_{22}H_{18}O_{11}$<br>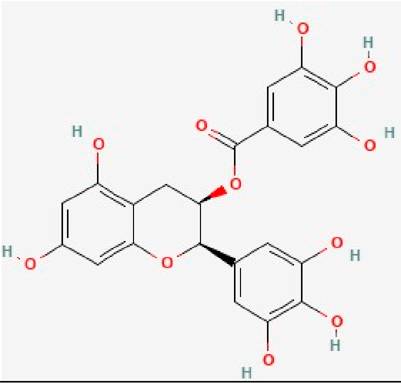
Fisetin<br>(528-48-3)	3,3′,4′,7-Tetrahydroxyflavone	286.236	$C_{15}H_{10}O_6$<br>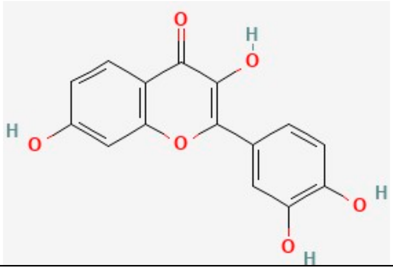
Galangin<br>(548-83-4)	3,5,7-Trihydroxyflavone	270.24	$C_{15}H_{10}O_5$<br>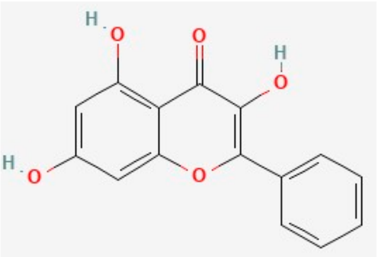

Gallocatechin (970-73-0)	(2*R*,3*S*)-2-(3,4,5-trihydroxy-phenyl)-3,4-dihydro-2H-chromene-3,5,7-triol	306.27	$C_{15}H_{14}O_7$ 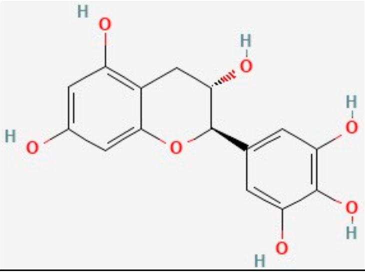
Genistein (446-72-0)	4′,5,7-Trihydroxyisoflavone	270.24	$C_{15}H_{10}O_5$ 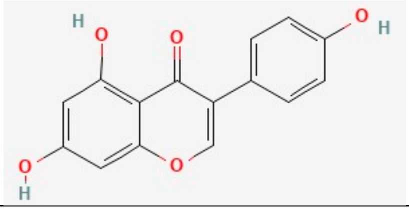
Hesperetin (520-33-2) (Hesperitin)	(2*S*)-5,7-dihydroxy-2-(3-hydroxy-4-methoxyphenyl)-2,3-dihydrochromen-4-one	302.28	$C_{16}H_{14}O_6$ 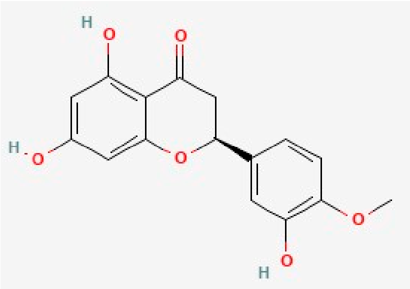
Isorhamnetin (3-methylquercetin) (480-19-3)	3,4′,5,7-Tetrahydoxy-3′-methoxyflavone	316.26	$C_{16}H_{12}O_7$ 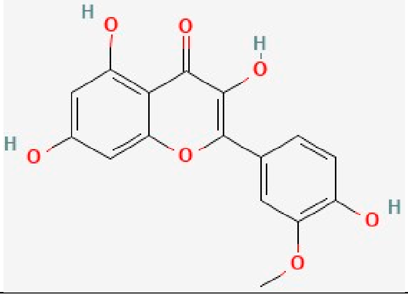
Kaempferide (491-54-3) (4′-Methoxylkaempferol)	3,5,7-trihydroxy-2-(4-methoxyphenyl)chromen-4-one	300.26	$C_{16}H_{12}O_6$ 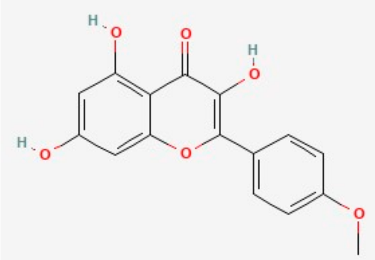

Kaempferol (520-18-3) (Kaempherol)	3,4′,5,7-Tetrahydroxyflavone	286.23	$C_{15}H_{10}O_6$ 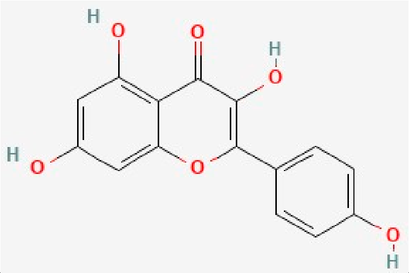
Karanjin (521-88-0)	3-methoxy-2-phenylfuro[2,3-h]chromen-4-one	292.3	$C_{18}H_{12}O_4$ 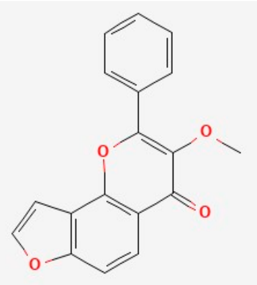
Karanjachromene (38070-93-8)	3-methoxy-8,8-dimethyl-2-phenylpyrano[2,3-f]chromen-4-one	334.4	$C_{21}H_{18}O_4$ 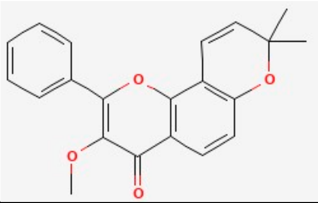
Luteolin (491-70-3)	3′,4′,5,7-Tetrahydroxyflavone	286.239	$C_{15}H_{10}O_6$ 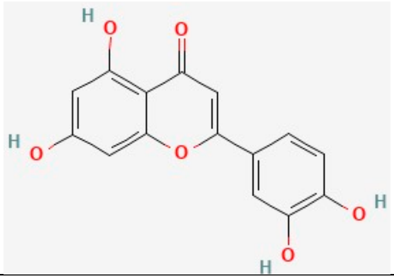
Morin (654055-01-3)	2′,3,4′,5,7-Pentahydroxyfla-vone	302.238	$C_{15}H_{10}O_7$ 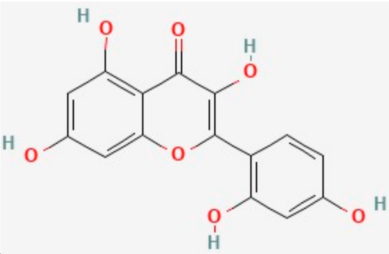

Myricetin (529-44-2)	3,3′,4′,5,5′,7-Hexahydroxyflavone	318.237	$C_{15}H_{10}O_8$ 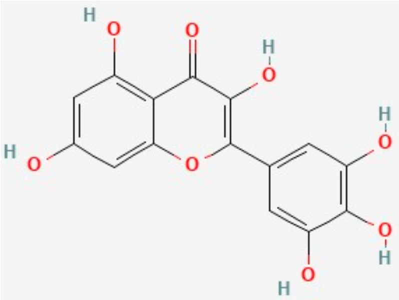
Naringenin (480-41-1)	(2*S*)-5,7-dihydroxy-2-(4-hydroxyphenyl)-2,3-dihydrochromen-4-one	272.25	$C_{15}H_{12}O_5$ 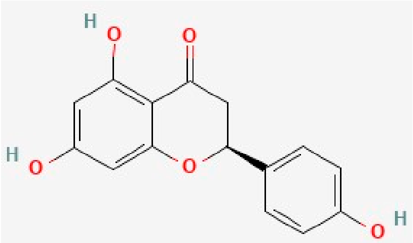
Pinobanksin (548-82-3) (3,5,7-Trihydroxyflavanone)	(2*R*,3*R*)-3,5,7-trihydroxy-2-phenyl-2,3-dihydrochromen-4-one	272.25	$C_{15}H_{12}O_5$ 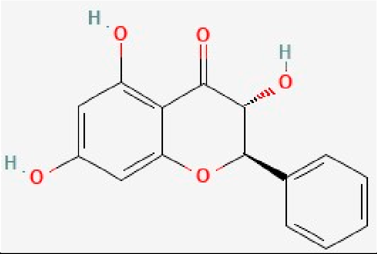
Pinocembrin (68745-38-0)	5,7-Dihydroxy-2-phehyl-2,3-dihydro-4H-chromen-4-one	256.257	$C_{15}H_{12}O_4$ 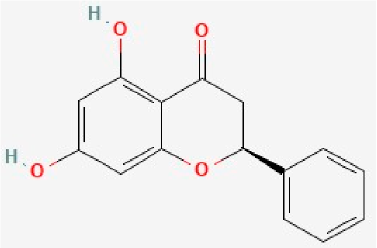
Quercetin (117-39-5)	3,3′,4′,5,7-Pentahydroxyflavone	302.236	$C_{15}H_{10}O_7$ 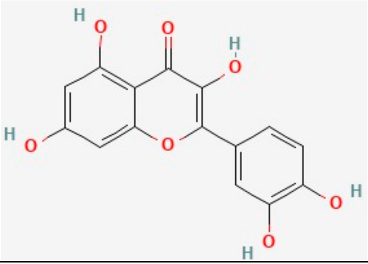
Taxifolin (480-18-2) (Dihydroquercetin)	(2*R*,3*R*)-3,3′,4′,5,7-Pentahydroxyflavan-4-one	304.254	$C_{15}H_{12}O_7$ 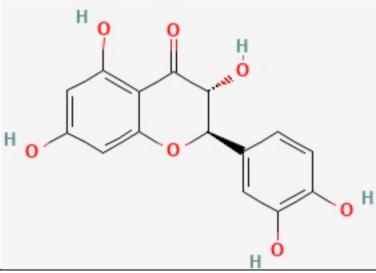

Tricin (520-32-1)	4′,5,7-Trihydroxy-3′,5′-dimethoxyflavone	330.29	$C_{17}H_{14}O_7$ 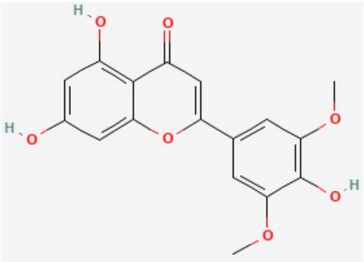

[1] Molecular weights and chemical structures were first retrieved from PubChem (https://pubchem.ncbi.nlm.nih.gov) on 2.14.2024.

Table S2. Flavonoid glycosides mentioned in this manuscript with common name, CAS number, IUPAC name, molecular weight, and chemical structure.[1] .

Common name (CAS number)	IUPAC name	Molecular weight (g/mol)	Chemical structure
Acetin 7-*O*-glucoside (?)	5-hydroxy-2-(4-methoxyphenyl)-7-[(2*S*,4*S*,5*S*)-3,4,5-trihydroxy-6-(hydroxymethyl)oxan-2-yl]oxychromen-4-one	446.4	$C_{22}H_{22}O_{10}$ 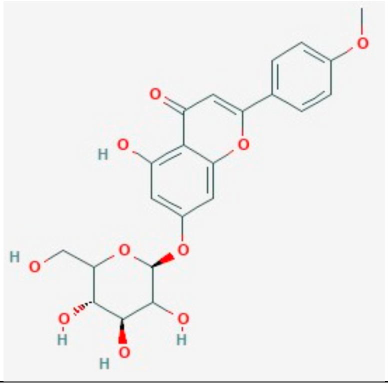
Afzelin (482-39-3) (Kaempferol 3-*O*-rhamnoside; Kaempferin)	5,7-dihydroxy-2-(4-hydroxyphenyl)-3-[(2*S*,3*R*,4*R*,5*R*,6*S*)-3,4,5-trihydroxy-6-methyloxan-2-yl]oxychromen-4-one	432.4	$C_{21}H_{20}O_{10}$ 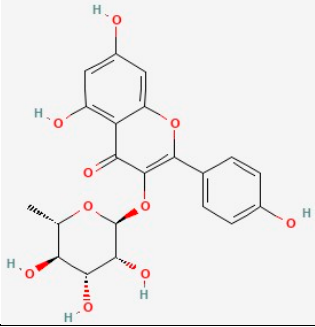
Astragalin (480-10-4) (Kaempferol 3-*O*-glucoside)	5,7-dihydroxy-2-(4-hydroxyphenyl)-3-[(2*S*,3*R*,4*S*,5*S*,6*R*)-3,4,5-trihydroxy-6-(hydroxymethyl)oxan-2-yl]oxychromen-4-one	448.4	$C_{21}H_{20}O_{11}$ 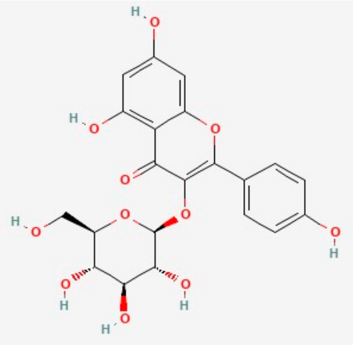

Catechin 7-*O*-β-glucoside (65597-47-9)	2-[[2-(3,4-dihydroxyphenyl)-3,5-dihydroxy-3,4-dihydro-2*H*-chromen-7-yl]oxy]-6-(hydroxymethyl)oxane-3,4,5-triol	452.4	$C_{21}H_{24}O_{11}$ 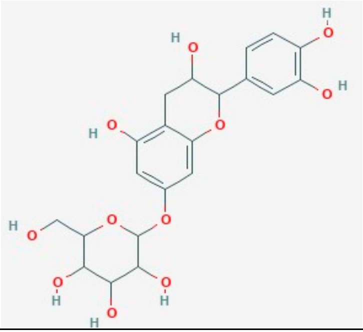
Catechin 7-xyloside (42830-48-8) (Catechin 7-*O*-β-D-xylopyranoside)	(2*S*,3*R*,4*S*,5*R*)-2-[[(2*R*,3*S*)-2-(3,4-dihydroxyphenyl)-3,5-dihydroxy-3,4-dihydro-2H-chromen-7-yl]oxy]oxane-3,4,5-triol	422.4	$C_{20}H_{22}O_{10}$ 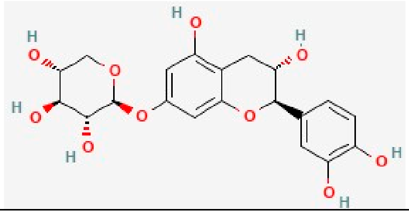
Cyanidin 3-glucoside (7084-24-4) (Asterin)	2-[2-(3,4-dihydroxyphenyl)-5,7-dihydroxychromenylium-3-yl]oxy-6-(hydroxymethyl)oxane-3,4,5-triol;chloride	484.8	$C_{21}H_{21}O_{11}$ 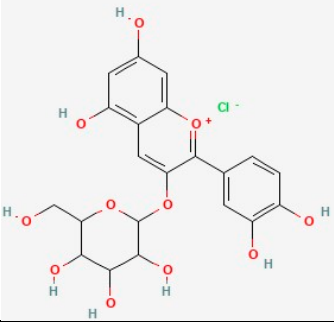
Hesperidin (520-26-3) (Hesperetin 7-*O*-rutinoside)	(2*S*)-5-hydroxy-2-(3-hydroxy-4-methoxyphenyl)-7-[(2*S*,3*R*,4*S*,5*S*,6*R*)-3,4,5-trihydroxy-6-[[(2*R*,3*R*,4*R*,5*R*,6*S*)-3,4,5-trihydroxy-6-methyloxan-2-yl]oxymethyl]oxan-2-yl]oxy-2,3-dihydrochromen-4-one	610.6	$C_{28}H_{34}O_{1}$ 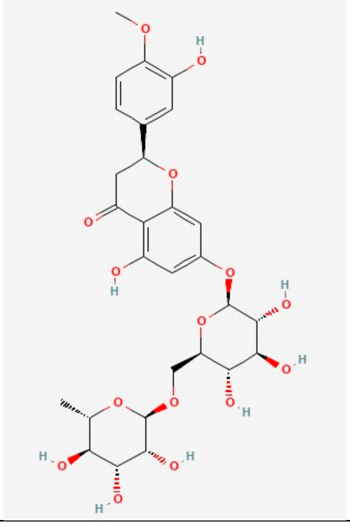

Hyperoside (482-36-0) (Hyperin, Quercetin 3-*O*-galactoside)	2-(3,4-dihydroxyphenyl)-5,7-dihydroxy-3-[(2*S*,3*R*,4*S*,5*R*,6*R*)-3,4,5-trihydroxy-6-(hydroxymethyl)oxan-2-yl]oxychromen-4-one	464.4	$C_{21}H_{20}O_{12}$ 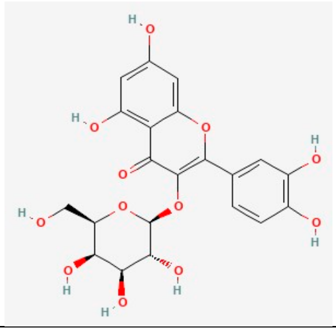
Isoquercetin (482-35-9) (Quercetin 3-*O*-glucoside, Isoquercitrin)	2-(3,4-dihydroxyphenyl)-5,7-dihydroxy-3-[(2*S*,3*R*,4*S*,5*S*,6*R*)-3,4,5-trihydroxy-6-(hydroxymethyl)oxan-2-yl]oxychromen-4-one	464.4	$C_{21}H_{20}O_{12}$ 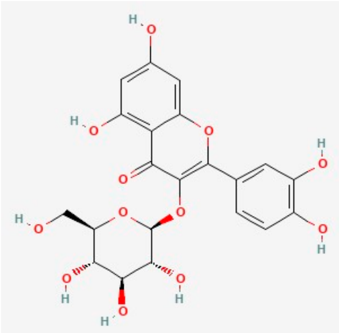
Isoorientin (4261-42-1) (Luteolin 6-*C*-glucoside)	2-(3,4-dihydroxyphenyl)-5,7-dihydroxy-6-[(2*S*,3*R*,4*R*,5*S*,6*R*)-3,4,5-trihydroxy-6-(hydroxymethyl)oxan-2-yl]chromen-4-one	448.4	$C_{21}H_{20}O_{11}$ 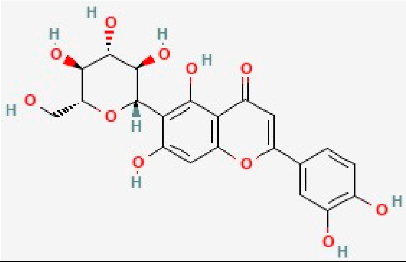
Isovitexin (38953-85-4) (Saponaretin)	5,7-dihydroxy-2-(4-hydroxyphenyl)-6-[(2*S*,3*R*,4*R*,5*S*,6*R*)-3,4,5-trihydroxy-6-(hydroxymethyl)oxan-2-yl]chromen-4-one	432.4	$C_{21}H_{20}O_{10}$ 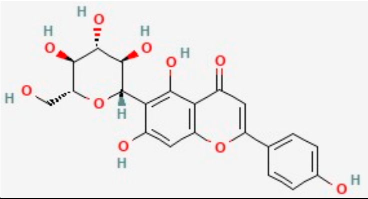
Kaempferol 3-*O*-sophoroside (19895-95-5)	3-[(2*S*,3*R*,4*S*,5*S*,6*R*)-4,5-dihydroxy-6-(hydroxymethyl)-3-[(2*S*,3*R*,4*S*,5*S*,6*R*)-3,4,5-trihydroxy-6-(hydroxymethyl)oxan-2-yl]oxyoxan-2-yl]oxy-5,7-dihydroxy-2-(4-hydroxyphenyl)chromen-4-one	610.5	$C_{27}H_{30}O_{16}$ 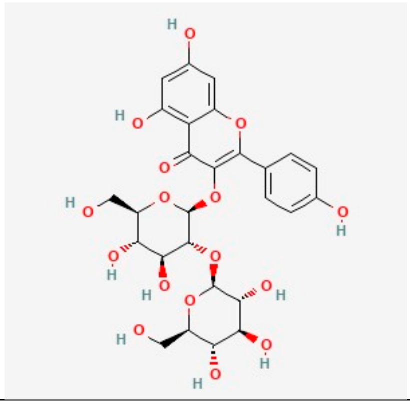

Kaempferol 3,7,4′-triglucoside (?) (Kaempferol 3,7,4′-tri-*O*-β-glucoside)	5-hydroxy-7-[(2*S*,4*S*,5*S*)-3,4,5-trihydroxy-6-(hydroxymethyl)oxan-2-yl]oxy-3-[(2*S*,5*S*)-3,4,5-trihydroxy-6-(hydroxymethyl)oxan-2-yl]oxy-2-[4-[(2*S*,4*S*,5*S*)-3,4,5-trihydroxy-6-(hydroxymethyl)oxan-2-yl]oxyphenyl]chromen-4-one	772.7	$C_{33}H_{40}O_{21}$ 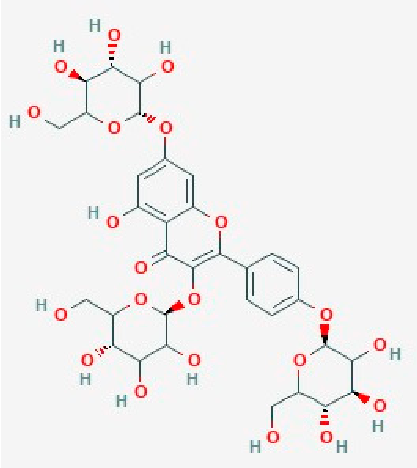
Luteolin 7-*O*-glucoside (5373-11-5) (Luteolin 7-glucoside, Luteoloside, Cynaroside)	2-(3,4-dihydroxyphenyl)-5-hydroxy-7-[(2*S*,3*R*,4*S*,5*S*,6*R*)-3,4,5-trihydroxy-6-(hydroxymethyl)oxan-2-yl]oxychromen-4-one	448.4	$C_{21}H_{20}O_{11}$ 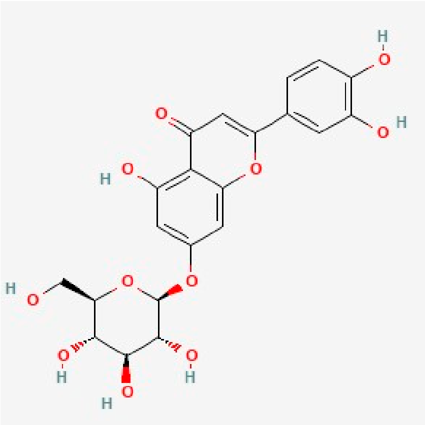
Luteolin 7-O-(6''-malonylglucoside) (98767-38-5) (Luteolin 7-O-β-D-(6''-O-malonyl) glucopyranoside)	3-[[(2*R*,3*S*,4*S*,5*R*,6*S*)-6-[2-(3,4-dihydroxyphenyl)-5-hydroxy-4-oxochromen-7-yl]oxy-3,4,5-trihydroxyoxan-2-yl]methoxy]-3-oxopropanoic acid	534.4	$C_{24}H_{22}O_{14}$ 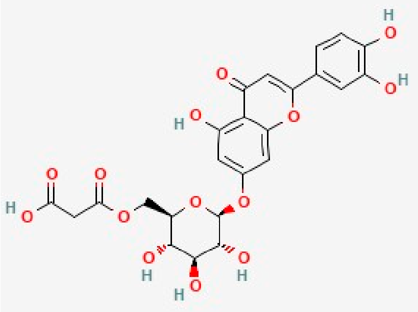
Myricetin 3-galactoside (15648-86-9)	5,7-dihydroxy-3-[(2*S*,3*R*,4*S*,5*R*,6*R*)-3,4,5-trihydroxy-6-(hydroxymethyl)oxan-2-yl]oxy-2-(3,4,5-trihydroxyphenyl)chromen-4-one	480.4	$C_{21}H_{20}O_{13}$ 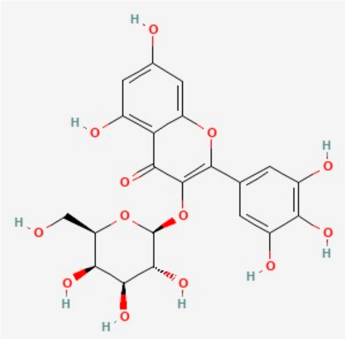

Myricetin 3-*O*-glucuronoside (77363-65-6) (Myricetin 3-*O*-β-D-glucuronide; Myricetin 3-*O*-glucuronide)	(2*S*,3*S*,4*S*,5*R*,6*S*)-6-[5,7-dihydroxy-4-oxo-2-(3,4,5-trihydroxyphenyl)chromen-3-yl]oxy-3,4,5-trihydroxyoxane-2-carboxylic acid	494.4	$C_{21}H_{18}O_{14}$ 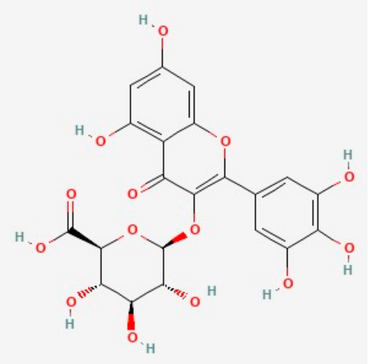
Myricitrin (17912-87-7) (Myricetin 3-*O*-rhamnoside)	5,7-dihydroxy-3-[(2*S*,3*R*,4*R*,5*R*,6*S*)-3,4,5-trihydroxy-6-methyloxan-2-yl]oxy-2-(3,4,5-trihydroxyphenyl)chromen-4-one	464.4	$C_{21}H_{20}O_{12}$ 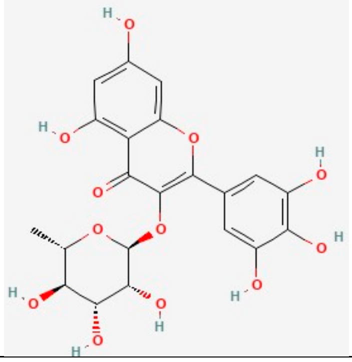
Naringin (10236-47-2) (Naringenin 7-*O*-neohesperidoside)	(2*S*)-7-[(2*S*,3*R*,4*S*,5*S*,6*R*)-4,5-dihydroxy-6-(hydroxymethyl)-3-[(2*S*,3*R*,4*R*,5*R*,6*S*)-3,4,5-trihydroxy-6-methyloxan-2-yl]oxyoxan-2-yl]oxy-5-hydroxy-2-(4-hydroxyphenyl)-2,3-dihydrochromen-4-one	580.5	$C_{27}H_{32}O_{14}$ 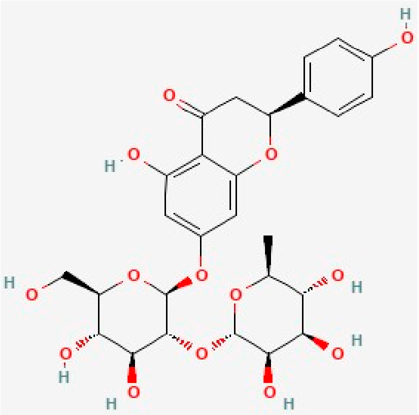

Narirutin (14259-46-2) (Naringenin 7-O-rutinoside)	(2S)-5-hydroxy-2-(4-hydroxyphenyl)-7-[(2S,3R,4S,5S,6R)-3,4,5-trihydroxy-6-[[(2R,3R,4R,5R,6S)-3,4,5-trihydroxy-6-methyloxan-2-yl]oxymethyl]oxan-2-yl]oxy-2,3-dihydrochromen-4-one	580.5	$C_{27}H_{32}O_{14}$ 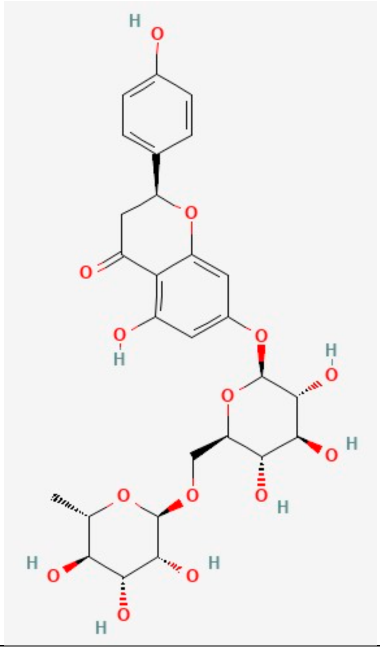
Neohesperidin (13241-33-3) (Hesperetin 7-*O*-neohesperidoside)	7-[4,5-dihydroxy-6-(hydroxymethyl)-3-(3,4,5-trihydroxy-6-methyloxan-2-yl)oxyoxan-2-yl]oxy-5-hydroxy-2-(3-hydroxy-4-methoxyphenyl)-2,3-dihydrochromen-4-one	610.6	$C_{28}H_{34}O_{15}$ 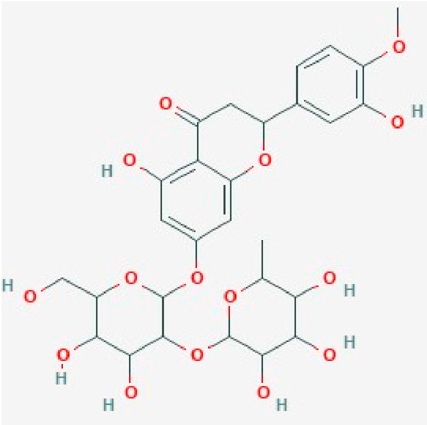
Nicotiflorin (17650-84-9) (Kaempferol 3-*O*-rutinoside)	5,7-dihydroxy-2-(4-hydroxyphenyl)-3-[(2*S*,3*R*,4*S*,5*S*,6*R*)-3,4,5-trihydroxy-6-[[(2*R*,3*R*,4*R*,5*R*,6*S*)-3,4,5-trihydroxy-6-methyloxan-2-yl]oxymethyl]oxan-2-yl]oxychromen-4-one	594.5	$C_{27}H_{30}O_{15}$ 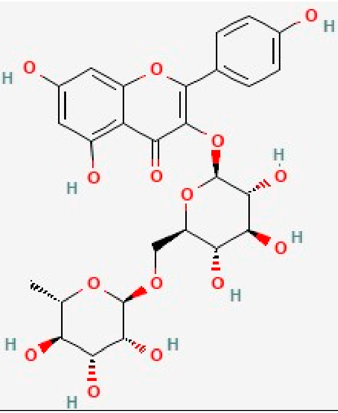

Orientin (28608-75-5) (Luteolin 8-*C*-glucoside)	2-(3,4-dihydroxyphenyl)-5,7-dihydroxy-8-[(2*S*,3*R*,4*R*,5*S*,6*R*)-3,4,5-trihydroxy-6-(hydroxymethyl)oxan-2-yl]chromen-4-one	448.4	$C_{21}H_{20}O_{11}$ 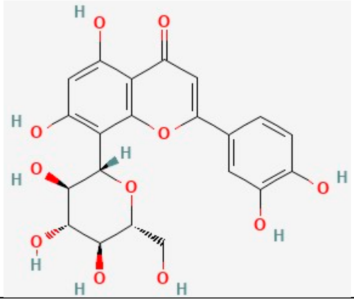
Phellamurin (52589-11-4) (Fellavine)	(2*R*,3*R*)-3,5-dihydroxy-2-(4-hydroxyphenyl)-8-(3-methylbut-2-enyl)-7-[(2*S*,3*R*,4*S*,5*S*,6*R*)-3,4,5-trihydroxy-6-(hydroxymethyl)oxan-2-yl]oxy-2,3-dihydrochromen-4-one	518.5	$C_{26}H_{30}O_{11}$ 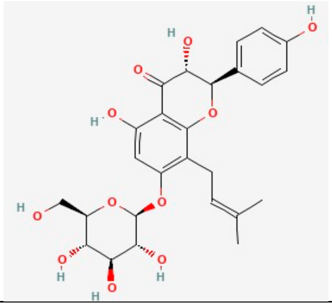
Poncirin (14941-08-3) (Citrifolioside)	(2*S*)-7-[(2*S*,3*R*,4*S*,5*S*,6*R*)-4,5-dihydroxy-6-(hydroxymethyl)-3-[(2*S*,3*R*,4*R*,5*R*,6*S*)-3,4,5-trihydroxy-6-methyloxan-2-yl]oxyoxan-2-yl]oxy-5-hydroxy-2-(4-methoxyphenyl)-2,3-dihydrochromen-4-one	594.6	$C_{28}H_{34}O_{14}$ 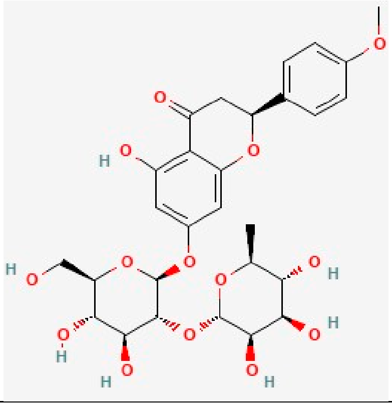
Quercetin 3′-glucoside (19254-30-9)	3,5,7-trihydroxy-2-[4-hydroxy-3-[(2*S*,3*R*,4*S*,5*S*,6*R*)-3,4,5-trihydroxy-6-(hydroxymethyl)oxan-2-yl]oxyphenyl]chromen-4-one	464.4	$C_{21}H_{20}O_{12}$ 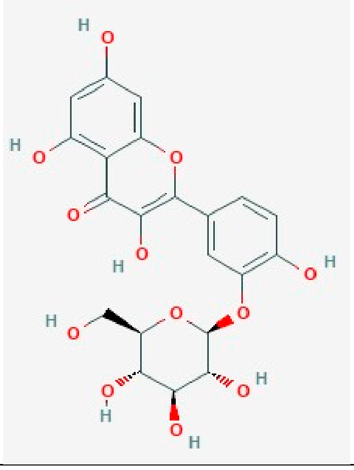
Quercetin 7-glucoside (491-50-9) (Quercimeritrin)	2-(3,4-dihydroxyphenyl)-3,5-dihydroxy-7-[(2*S*,3*R*,4*S*,5*S*,6*R*)-3,4,5-trihydroxy-6-(hydroxymethyl)oxan-2-yl]oxychromen-4-one	464.4	$C_{21}H_{20}O_{12}$

			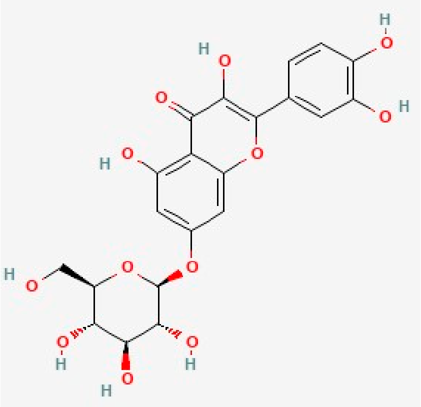
Quercetin 3-*O*-glucuronoside (22688-79-5) (Miquelianin; Quercetin 3-*O*-glucuronide; Querciturone)	(2*S*,3*S*,4*S*,5*R*,6*S*)-6-[2-(3,4-dihydroxyphenyl)-5,7-dihydroxy-4-oxochromen-3-yl]oxy-3,4,5-trihydroxyoxane-2-carboxylic acid	478.4	$C_{21}H_{18}O_{13}$ 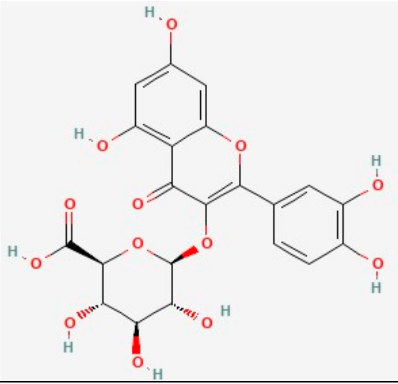
Quercetin 3,7,4′-triglucoside (133563-23-2) (Quercetin 3,7,4′-tri-*O*-β-glucoside)	5-hydroxy-2-[3-hydroxy-4-[(2S,5S)-3,4,5-trihydroxy-6-(hydroxymethyl)oxan-2-yl]oxyphenyl]-3-[(2S,5S)-3,4,5-trihydroxy-6-(hydroxymethyl)oxan-2-yl]oxy-7-[(2S,5S,6S)-3,4,5-trihydroxy-6-(hydroxymethyl)oxan-2-yl]oxychromen-4-one	788.7	$C_{33}H_{40}O_{22}$ 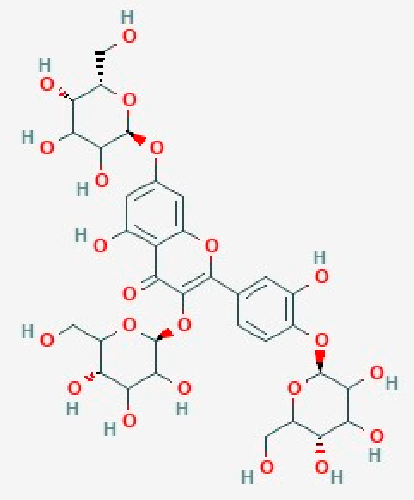
Quercitrin (522-12-3) (Quercetin 3-*O*-rhamnoside; Quercetrin)	2-(3,4-dihydroxyphenyl)-5,7-dihydroxy-3-[(2*S*,3*R*,4*R*,5*R*,6*S*)-3,4,5-trihydroxy-6-methyloxan-2-yl]oxychromen-4-one	448.4	$C_{21}H_{20}O_{11}$ 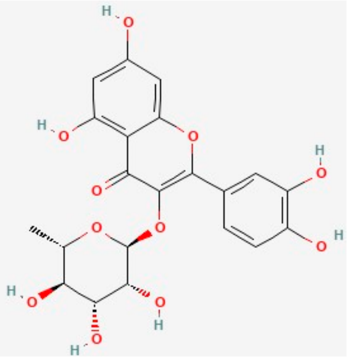

Rhoifolin (17306-46-6) (Apigenin 7-*O*-neohesperidoside; Rhoifoloside)	7-[(2*S*,3*R*,4*S*,5*S*,6*R*)-4,5-dihydroxy-6-(hydroxymethyl)-3-[(2*S*,3*R*,4*R*,5*R*,6*S*)-3,4,5-trihydroxy-6-methyloxan-2-yl]oxyoxan-2-yl]oxy-5-hydroxy-2-(4-hydroxyphenyl)chromen-4-one	578.5	$C_{27}H_{30}O_{14}$ 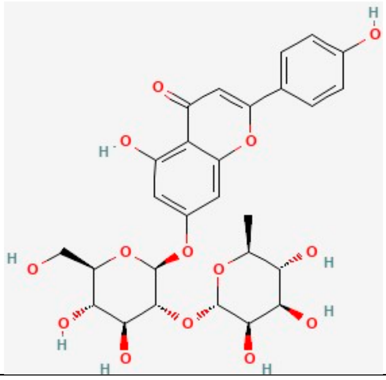
Rutin (153-18-4) (Quercetin 3-*O*-rutinoside)	2-(3,4-dihydroxyphenyl)-5,7-dihydroxy-3-[(2*S*,3*R*,4*S*,5*S*,6*R*)-3,4,5-trihydroxy-6-[[(2*R*,3*R*,4*R*,5*R*,6*S*)-3,4,5-trihydroxy-6-methyloxan-2-yl]oxymethyl]oxan-2-yl]oxychromen-4-one	610.5	$C_{27}H_{30}O_{16}$ 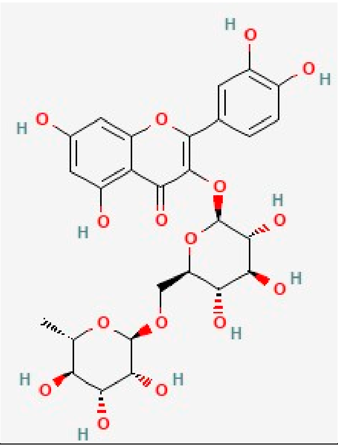
Taxifolin 3-glucoside (27297-45-6) (Taxifolin 3-*O*-β-D-glucopyranoside)	(2*R*,3*R*)-2-(3,4-dihydroxyphenyl)-5,7-dihydroxy-3-[(2*S*,3*R*,4*S*,5*S*,6*R*)-3,4,5-trihydroxy-6-(hydroxymethyl)oxan-2-yl]oxy-2,3-dihydrochromen-4-one	466.4	$C_{21}H_{22}O_{12}$ 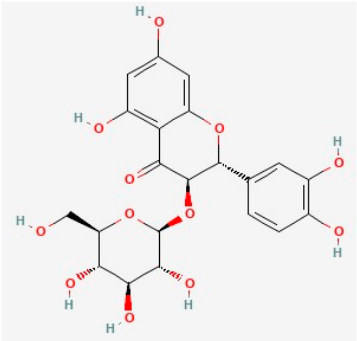
Vicenin-2 (23666-13-9) (Vicenin II; Isovitexin 8-*C*-beta-glucoside)	5,7-dihydroxy-2-(4-hydroxyphenyl)-6,8-bis[(2*S*,3*R*,4*R*,5*S*,6*R*)-3,4,5-trihydroxy-6-(hydroxymethyl)oxan-2-yl]chromen-4-one	594.5	$C_{27}H_{30}O_{15}$ 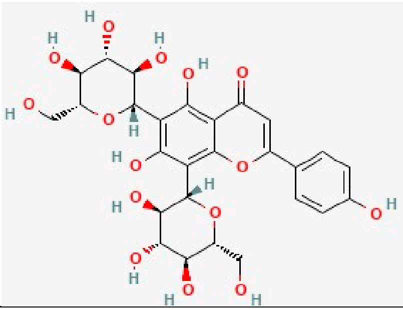

Vitexin (3681-93-4) (Apigenin 8-*C*-glucoside)	5,7-dihydroxy-2-(4-hydroxyphenyl)-8-[(2*S*,3*R*,4*R*,5*S*,6*R*)-3,4,5-trihydroxy-6-(hydroxymethyl)oxan-2-yl]chromen-4-one	432.4	$C_{21}H_{20}O_{10}$ 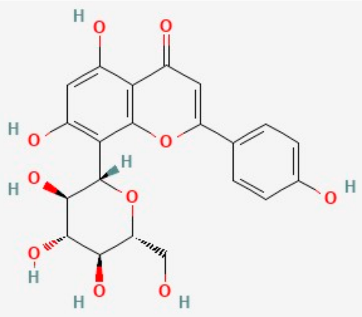

[1] Molecular weights and chemical structures were first retrieved from PubChem (https://pubchem.ncbi.nlm.nih.gov) on 2.15.2024.

Table S3. Flavonoids found to have positive (++), negative (--), or neutral (o) effects on insect behavior and life history parameters, with references.[1]

Common name	Taxa	Parameters [1]	References
Acacetin	Sawflies	Development (--)	[108]
Apigenin	Butterflies	Sequestration (o)	[53]
	Beetles	Feeding (++)	[135]
	True Bugs	Feeding (--)	[173]
Aromadendrin	Beetles	Feeding (++)	[141]
(+)-Catechin	Sawflies	Cocoon mass (--), high conc.	[104]
	Beetles	Oviposition, in part (++)	[110]
	Beetles	Feeding (--), high conc.	[149]
	Beetles	Feeding (--), high conc.	[148]
	Beetles	Feeding and tunneling (--)	[112]
	Beetles	Degradation of host defenses (++)	[114]
	Beetles	Oviposition (++)	[127]
	Beetles	Oviposition (++)	[124]
	True Flies	Egg Hatch (--), high conc.	[194]
	True Flies	Development (--), high conc.	[194]
Chrysin	Bees	Foraging capacity (--), low conc.	[70]
	Bees	Feeding (o)	[70]
	True Flies	Development (--), high conc.	[200]
	True Flies	Pupation (--), high conc.	[200]
	True Flies	Emergence (--), high conc.	[200]
	True Flies	Oviposition (--), Fecundity (o)	[200]
Daidzein	True Bugs	Feeding (--)	[173]
	True Flies	Survival (--)	[186]
	True Flies	Enzyme activity (--)	[187]
Desmethylglycitein (DMG)	True Flies	Enzyme activity (--), Development (--), Survival (--)	[187] [187]
Epigallocatechin gallate (EGCG)	True Bugs	Survival (--)	[182]
Fisetin	Beetles	Body mass (--), Survival (o)	[128]
Galangin	Bees	Foraging capacity (o), Feeding (o)	[70]
Gallocatechin	Beetles	Host defenses (--)	[114]
Genistein	True Bugs	Survival (o), *Medicago* host race	[172]
	True Bugs	Survival (--), *Pisum* host race	[172]

	True Bugs	Feeding (o)	[173]
	True Bugs	Feeding (--), high conc.	[171]
	True Flies	Survival (--)	[186]
Hesperetin	Beetles	Feeding (++)	[135]
Isorhamnetin	Beetles	Feeding (++)	[135]
	True Bugs	Fecundity (--)	[167]
Kaempferide	Sawflies	Development (--)	[108]
Kaempferol	Butterflies	Sequestration (o)	[56]
	Bees	Survival (++)	[75]
	Beetles	Feeding (o)	[122]
	Beetles	Feeding (o)	[148]
	True Bugs	Fecundity (o)	[167]
	True Bugs	Feeding (--)	[173]
Karanjin	True Flies	Survival (--)	[188]
Karanjachromene	True Flies	Survival (--)	[188]
Luteolin	Butterflies	Sequestration (o)	[53]
	Beetles	Feeding (o)	[129]
	True Bugs	Feeding (--), high conc.	[171]
Morin	Beetles	Feeding (++)	[131]
Myricetin	Beetles	Feeding (++)	[131]
	Beetles	Body mass (--)	[128]
	Beetles	Survival (o)	[128]
Naringenin	Bees	Foraging capacity (o)	[70]
	Bees	Feeding (++), at high conc.	[70]
	Beetles	Feeding (--)	[141]
	Beetles	Feeding (--), high conc.	[149]
	Beetles	Feeding (--), high conc.	[148]
	Beetles	Feeding (++)	[135]
	Beetles	Oviposition (--)	[159]
	True Bugs	Development (--), high conc.	[168]
	True Bugs	Fecundity (--), high conc.	[168]
	True Bugs	Survival (--), high conc.	[168]
	True Flies	Egg Hatch (--), high conc.	[194]
	True Flies	Development (--), high conc.	[194]
Pinobanksin	Bees	P450s enzyme activity (++)	[66]
	Bees	Pesticide detox. (++), in part	[66]
Pinocembrin	Bees	P450s enzyme activity (++)	[66]
	Bees	Pesticide detox. (++), in part	[66]
	Bees	Foraging capacity (++), low conc.	[70]
	Bees	Feeding (o)	[70]
	Beetles	Feeding (++)	[141]
	Beetles	Feeding (--), Development (--),	[154]
	Beetles	Survival (--)	[154]
Quercetin	Butterflies	Sequestration (o)	[56]
	Butterflies	Enzyme activity (++)	[25]
	Butterflies	Enzyme activity (--)	[25]
	Butterflies	Enzyme activity (--)	[25]
	Bees	P450s enzyme activity (++)	[64]
	Bees	P450s enzyme activity (++)	[65]
	Bees	P450s enzyme activity (++)	[66]
	Bees	Pesticide detox. (++), in part	[65]
	Bees	Energy production (--)	[69]

	Bees	Ovarian development (++)	[83]
	Bees	Survival (--)	[84]
	Bees	Foraging capacity (++)	[70]
	Bees	Lifespan (++)	[71]
	Bees	Wing beat frequency (++),	[73]
	Bees	Energy production (++)	[73]
	Bees	Lifespan (++)	[72]
	Bees	Survival (++), low conc.	[74]
	Bees	Pesticide detox. (++)	[85]
	Bees	Pesticide conc. (o)	[85]
	Bees	Pesticide detox. (++), low conc.	[90]
	Bees	Visual attractant (o)	[95]
	Beetles	Body mass (++), low conc.	[121]
	Beetles	Oviposition (o), Feeding (o)	[121]
	Beetles	Feeding (++)	[122]
	Beetles	Feeding (--)	[141]
	Beetles	Feeding (++)	[131]
	Beetles	Survival (--)	[136]
	Beetles	Feeding (++)	[138]
	Beetles	Oviposition (o)	[124]
	Beetles	Feeding (--), high conc.	[149]
	Beetles	Feeding (++), high conc.	[148]
	Beetles	Oviposition (++)	[159]
	Beetles	Oviposition (++)	[160]
	Beetles	Feeding (--)	[144]
	Beetles	Feeding (o), Development (o)	[154]
	Beetles	Survival (--), high conc.	[154]
	Beetles	Oviposition (--), Body mass (--)	[128]
	Beetles	Emergence (--), Survival (--)	[128]
	Beetles	Feeding (o)	[150]
	True Bugs	Fecundity (--), high conc.	[168]
	True Bugs	Survival (--), high conc.	[168]
	True Bugs	Development (--), high conc.	[168]
	True Bugs	Fecundity (--)	[167]
	True Bugs	Feeding (--), high conc.	[174]
	True Bugs	Survival (--), high conc.	[179]
	True Flies	Survival (--)	[190]
	True Flies	Egg Hatch (--), high conc.	[194]
	True Flies	Development (--), high conc.	[194]
	True Flies	Survival (--)	[191]
	True Flies	Chromosome segregation (o)	[193]
	True Flies	Fecundity (++)	[193]
	True Flies	Development (++)	[196]
	True Flies	Egg hatch (--), high conc.	[197]
	True Flies	Body wt. (--), high conc.	[197]
	True Flies	Emergence (--), high conc.	[197]
	True Flies	Oviposition (--), high conc.	[199]
Taxifolin	Beetles	Feeding (++)	[141]
	Beetles	Feeding and tunneling (--)	[112]
	Beetles	Oviposition (++)	[124]
	Beetles	Oviposition (++)	[159]
	Beetles	Feeding (--)	[135]
	Beetles	Survival (--)	[136]

Tricin	Butterflies	Sequestration (o)	[53]

[1] Refer to the manuscript text for information on the insect species tested.

Table S4. Flavonoid glycosides found to have positive (++), negative (--), or neutral (o) effects on insect behavior and life history parameters, with references.[1]

Common name	Taxa	Parameters	References
Acetin 7-*O*-glucoside	Sawflies	Metabolism (o)	[106]
Afzelin	Butterflies	Sequestration/Mating (++)	[47]
Astragalin	Butterflies	Sequestration/Mating (++)	[45]
	Sawflies	Development (o)	[106]
(+)-Catechin 7-*O*-β-glucoside	Sawflies	Sequestration (o)	[105]
(+)-Catechin 7-xyloside	Beetles	Feeding behavior (++), with lupeyl cerotate	[117]
Cyanidin 3-glucoside	Beetles	Feeding (o)	[122]
Hesperidin	Butterflies	Oviposition (++)	[35]
	Butterflies	Oviposition (++)	[39]
	Butterflies	Oviposition (++)	[40]
Hyperoside	Butterflies	Sequestration/Mating (++)	[45]
	Sawflies	Cocoon mass (o)	[104]
	Sawflies	Sequestration/Defense (++)	[98]
Isoquercetin	Butterflies	Sequestration (o)	[33]
	Sawflies	Sequestration/Defense (++)	[98]
	Beetles	Feeding (o)	[122]
Isoorientin	Butterflies	Sequestration (o)	[53]
	Beetles	Feeding (--), Oviposition (--)	[123]
Isovitexin	Beetles	Feeding (--), Oviposition (--)	[123]
Kaempferol 3-*O*-sophoroside	Butterflies	Sequestration (o)	[51]
Kaempferol 3,7,4′-triglucoside	Sawflies	Development (o)	[105]
	Sawflies	Sequestration (o)	[105]
Luteolin 7-*O*-glucoside	Beetles	Feeding (++)	[129]
Luteolin 7-O-(6''-malonylglucoside)	Butterflies	Oviposition, in part (++)	[26]
Myricetin 3-galactoside	Sawflies	Cocoon mass (--), high conc.	[104]
Myricetin 3-*O*-glucuronoside	Sawflies	Development (o)	[106]
Myricitrin	Beetles	Feeding (++)	[131]
Naringin	Butterflies	Oviposition (++)	[39]
	Butterflies	Oviposition (++)	[40]
	True Bugs	Survival (--)	[179]
	True Flies	Oviposition (--), Egg Hatch (--)	[189]
	True Flies	Biting (--), Survival (--)	[189]
Narirutin	Butterflies	Oviposition (++)	[35]
Neohesperidin	Butterflies	Oviposition (o)	[40]
Nicotiflorin	Butterflies	Oviposition (--), high conc.	[30]
Orientin	Butterflies	Sequestration (o)	[53]
Phellamurin	Butterflies	Oviposition (--)	[41]
	Butterflies	Oviposition (--)	[42]
	Butterflies	Oviposition (++)	[42]
Poncirin	True Flies	Survival (--), Oviposition (--),	[189]
	True Flies	Egg hatch (--), Biting (--)	[189]
Quercetin 3′-glucoside	Beetles	Feeding (++)	[122]

Quercetin 7-glucoside	Beetles	Feeding (++)	[122]
Quercetin 3-*O*-glucuronoside	Sawflies	Development (o)	[106]
Quercetin 3,7,4′-triglucoside	Sawflies	Sequestration (o)	[105]
Quercitrin	Butterflies	Sequestration/Mating (++)	[47]
	Sawflies	Cocoon mass (o)	[104]
	Sawflies	Sequestration/Defense (++)	[98]
	Beetles	Feeding (++)	[131]
	Beetles	Body mass (--), Survival (o)	[128]
	True Bugs	Feeding (--)	[183]
Rhoifolin	True Flies	Survival (--), Oviposition (--)	[189]
	True Flies	Egg Hatch (--), Biting (--)	[189]
Rutin	Butterflies	Oviposition (--), high conc.	[30]
	Butterflies	Oviposition (++)	[35]
	Bees	Pesticide detox. (++)	[96]
	Sawflies	Sequestration/Defense (++)	[98]
	Beetles	Oviposition (o)	[121]
	Beetles	Body mass (o), Feeding (o)	[121]
	Beetles	Feeding (++)	[131]
	Beetles	Feeding (++), high conc.	[138]
	Beetles	Feeding (++), high conc.	[149]
	Beetles	Body mass (--), Survival (o)	[128]
	Beetles	Feeding (++), high conc.	[148]
	Beetles	Feeding (o)	[150]
	True Bugs	Growth (--), high conc.	[166]
	True Bugs	Survival (--), high conc.	[179]
	True Bugs	Feeding (--), high conc.	[174]
Taxifolin 3-glucoside	Sawflies	Body mass (o), Survival (o), Devel-	[101]
	Sawflies	opment (--)	[101]
Vicenin-2	Butterflies	Oviposition (++)	[35]
Vitexin	Butterflies	Sequestration (o)	[53]
	Beetles	Feeding (--), Oviposition (--)	[123]

[1] Refer to the manuscript text for information on the insect species tested. Any un-named flavonoid glycosides, such as quercetin glycoside, are not listed in this table.
